# Mutations in Conserved Residues of the *C. elegans* microRNA Argonaute ALG-1 Identify Separable Functions in ALG-1 miRISC Loading and Target Repression

**DOI:** 10.1371/journal.pgen.1004286

**Published:** 2014-04-24

**Authors:** Anna Y. Zinovyeva, Samir Bouasker, Martin J. Simard, Christopher M. Hammell, Victor Ambros

**Affiliations:** 1 Program in Molecular Medicine, University of Massachusetts Medical School, Worcester, Massachusetts, United States of America; 2 St-Patrick Research Group in Basic Oncology, Hôtel-Dieu de Québec (Centre Hospitalier Universitaire de Québec), Laval University Cancer Research Centre, Quebec City, Québec, Canada; 3 Cold Spring Harbor Laboratory, Cold Spring Harbor, New York, United States of America; University of California Riverside, United States of America

## Abstract

microRNAs function in diverse developmental and physiological processes by regulating target gene expression at the post-transcriptional level. ALG-1 is one of two *Caenorhabditis elegans* Argonautes (ALG-1 and ALG-2) that together are essential for microRNA biogenesis and function. Here, we report the identification of novel antimorphic (anti) alleles of ALG-1 as suppressors of *lin-28(lf)* precocious developmental phenotypes. The *alg-1(anti)* mutations broadly impair the function of many microRNAs and cause dosage-dependent phenotypes that are more severe than the complete loss of ALG-1. ALG-1(anti) mutant proteins are competent for promoting Dicer cleavage of microRNA precursors and for associating with and stabilizing microRNAs. However, our results suggest that ALG-1(anti) proteins may sequester microRNAs in immature and functionally deficient microRNA Induced Silencing Complexes (miRISCs), and hence compete with ALG-2 for access to functional microRNAs. Immunoprecipitation experiments show that ALG-1(anti) proteins display an increased association with Dicer and a decreased association with AIN-1/GW182. These findings suggest that *alg-1(anti)* mutations impair the ability of ALG-1 miRISC to execute a transition from Dicer-associated microRNA processing to AIN-1/GW182 associated effector function, and indicate an active role for ALG/Argonaute in mediating this transition.

## Author Summary

microRNAs are small non-coding RNAs that function in diverse processes by post-transcriptionally regulating gene expression. Argonautes form the core of the microRNA Induced Silencing Complex (miRISC) and are required for microRNA biogenesis and function. Here we describe the identification and characterization of a novel set of mutations in *alg-1*, a *Caenorhabditis elegans* microRNA specific Argonaute. This new class of *alg-1* mutations causes phenotypes more severe than the complete loss of *alg-1*. Interestingly, the mutant ALG-1 proteins are able to promote microRNA biogenesis, but are defective in mediating microRNA target gene repression. We found that mutant ALG-1 associates more with Dicer, but less with miRISC effector AIN-1, compared to wild type ALG-1. We propose that these mutant ALG-1 proteins assemble nonfunctional complexes that effectively compete with the paralogous ALG-2 for critical miRISC components, including mature microRNAs. This new class of Argonaute mutants highlights the role of Argonaute in mediating a functional transition for miRISC from microRNA processing phase to target repression phase.

## Introduction

Development of complex organisms requires execution of molecular and cellular events with precise temporal control. Impaired developmental timing can result in severe morphological abnormalities. In *C. elegans*, the heterochronic gene network controls the stage-specific execution of larval developmental events. *C. elegans* heterochronic mutants display either precocious or retarded development, due to skipping or reiteration of certain stage-specific cell fate programs. The heterochronic regulatory network consists of transcriptional regulatory proteins, RNA binding proteins, and microRNAs that regulate the developmental expression of those proteins (reviewed in [1]). In particular, the *let-7-Family* microRNAs, *let-7*, *mir-241*, *mir-48*, and *mir-84*, are key players in the heterochronic genetic network that controls progression through the *C. elegans* larval development [1]–[3]. Deletion of the *let-7* microRNA or its sisters *miR-48, miR-241 and miR-84* results in reiteration of larval stage-specific cell fates, delaying the adoption of adult cell fates [1]–[3].

microRNAs bind to target mRNAs via imperfect base pairing, bringing along the microRNA Induced Silencing Complex (miRISC), which in turn acts to destabilize the target mRNA and/or repress its translation (reviewed in [4]–[7]). microRNAs associate with Argonaute proteins (in *C. elegans*, ALG-1 or ALG-2), forming the core of the miRISC. Argonautes are not only critical for the activity of mature microRNAs in regulating their targets, but also function in conjunction with the ribonuclease Dicer (DCR-1 in *C. elegans*) in processing of microRNA hairpin precursors into mature microRNAs [8], [9] (reviewed in [10]). Loss of *alg-1* or depletion of *alg-1*/*alg-2* or *dcr-1* via RNA interference (RNAi) results in accumulation of microRNA precursors, consistent with ALG-1/2 and Dicer acting together at the microRNA biogenesis step of miRISC maturation [8], [9]. Upon miRISC maturation, ALG-1/ALG-2 form miRISC complexes with the effector proteins, including the GW182 homologs, AIN-1/AIN-2 [11], [12]. AIN-1/2 proteins are critical components of the distinct effector miRISC that functions on the mRNA targets to mediate mRNA degradation and/or translational repression [11]–[17]. However, while some recent data brought new insights about the contribution of Argonautes in the miRISC maturation process [8], little is known about the transition from the DCR-1-containing microRNA-processing complex to the GW182-containing effector complex.

**Figure 1:**
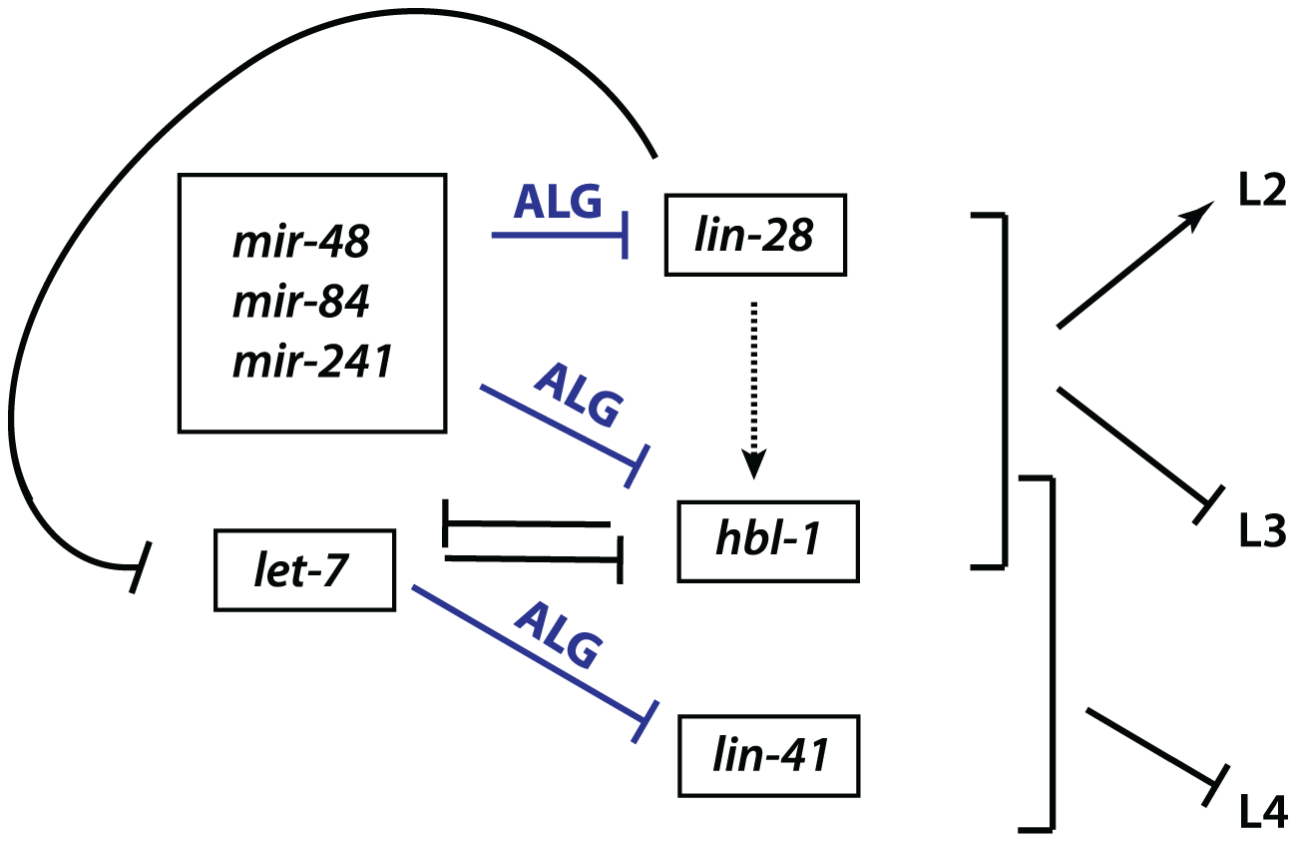
Genetic network of heterochronic genes regulating the developmental timing of cell divisions associated with the larval stages of *C. elegans* development. Based on [1].

**Figure 2:**
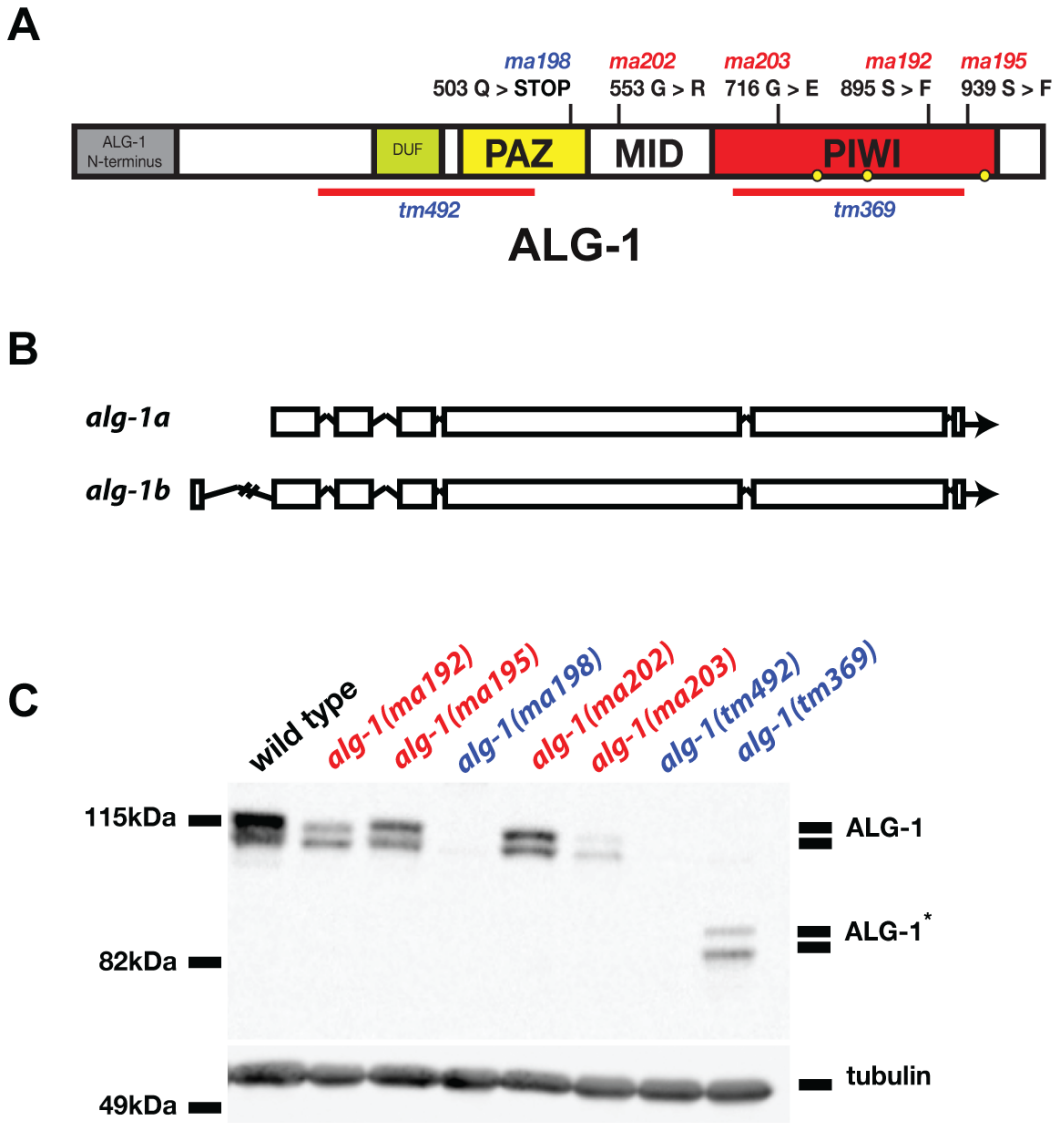
Four of five newly isolated *alg-1* alleles carry missense mutations. (**A**) A schematic showing the positions of the newly identified *alg-1* mutations within the ALG-1 protein (black tics) and the existing deletion alleles (red lines). Positions of catalytic sites are indicated by yellow circles. (**B**) Exon/intron schematic of the two *alg-1* isoforms predicted and supported by cDNA evidence (Wormbase.org). Boxes represent exonic regions. (**C**). Western blot analysis on total protein lysate from wild type and *alg-1* mutant animals. All non-null *alg-1* alleles, like the wild type, produce 2 isoforms of ALG-1. Newly identified missense alleles of *alg-1* are marked in red and null alleles are in blue. *alg-1(tm369)* is a loss of function allele that deletes most of the PIWI domain and produces 2 truncated isoforms of ALG-1(*). All strains with the exception of *alg-1(tm492)* and *alg-1(tm369)* carry *lin-31(lf)* and *col-19::gfp* in the background.

LIN-28 is an evolutionarily conserved RNA-binding protein that acts in opposition to *let-7-Family* microRNAs. *C. elegans lin-28(lf)* mutants skip developmental programs of the second (L2) and occasionally the third (L3) larval stages. Consequently, *lin-28(lf)* mutants execute adult-specific terminal differentiation one to two stages earlier than normal [18], [19]. By contrast, loss of *let-7-sisters (mir-241*, *mir-48*, and *mir-84)* results in the reiteration of L2 fates [1], [2], and loss of *let-7* causes the reiteration of L3 fates [1]–[3], [20]. *lin-28* promotes early cell fates via two distinct mechanisms (Figure 1). First, LIN-28 controls *let-7* microRNA levels by binding to the *let-7* pre-microRNA and facilitating its active turnover, a role first described in mammals [1]–[3], [21]–[28]. Second, *lin-28* positively regulates expression of *hbl-1* (Figure 1), [4]–[7], [20]. HBL-1 is a zinc-finger transcription factor that normally functions to repress the cell fates associated with the third larval stage (L3) [8], [9], [29], [30], and has been shown to inhibit transcription of *let-7* [8], [9], [31]. In turn, *hbl-1* is down regulated by the *let-7-Family* microRNAs prior to the third larval stage to allow the progression from the L2 to L3 to occur (Figure 1), [2], [11], [12], [29], [30]. In addition, *lin-28* itself is a predicted target for *let-7-Family* microRNAs [2], [11]–[17], [20], [32]. Thus, *lin-28*, *hbl-1* and *let-7-Family* microRNAs function in a complex regulatory network that controls the progression of *C. elegans* larval development (Figure 1), (reviewed by [1], [8], [33]).

With the goal of identifying microRNA co-factors that normally positively influence microRNA function, we took advantage of the opposing relationship between *lin-28* and *let-7-Family* microRNAs and performed a forward genetic screen for suppressors of a hypomorphic allele of *lin-28(lf)*. From this screen, we identified novel alleles in one of the two *C. elegans* microRNA Argonautes, ALG-1. Here, we report the unusual antimorphic properties of these mutations, which highlight the importance of the ALG-1 in miRISC maturation from a microRNA-processing complex to an effector complex. We interpret the nature of these alleles to reflect distinct and separable ALG-1 functions in microRNA biogenesis versus mRNA target repression.

## Results

### A *lin-28(lf)* suppressor screen yields mutations in the *C. elegans* microRNA specific Argonaute ALG-1

The highly penetrant developmental defects of *lin-28(lf)* mutants are in large part the consequence of elevated activity of certain microRNAs, principally *let-7* and other developmental timing microRNAs [20], [32]. Therefore, to identify factors that function in conjunction with *let-7-Family* microRNAs, we performed a forward genetic screen (estimated 39,000 EMS mutagenized genomes) for suppressors of *lin-28(lf)* phenotypes. *lin-28(lf)* animals are 100% egg laying defective, owing to an abnormal vulval morphology that results from their precocious cell lineage defects [18], [34]. Genetic suppressors of *lin-28(lf)* were identified by restoration of egg laying. Six of the eleven suppressor mutations were alleles of *lin-46*, a heterochronic gene previously shown to function downstream of *lin-28* [35]. The other five suppressor mutations mapped to the X chromosome and displayed a range of suppression of the *lin-28(lf)* heterochronic phenotypes. All five of these X linked suppressor mutations were determined to be alleles of *alg-1*, a gene that encodes one of two *C. elegans* microRNA specific Argonautes.

### Four of five *alg-1* novel alleles are missense mutations that permit accumulation of full length ALG-1 protein

Four of the five *alg-1* alleles isolated as *lin-28(lf)* suppressors (*ma192*, *ma195*, *ma202*, and *ma203*) are missense mutations (Figure 2A). The fifth mutation, *alg-1(ma198)* is a predicted null allele of *alg-1* (Figure 2A) and genetically behaves similarly to previously isolated null allele *alg-1(tm492)* (Figure 2A, Table 1, and data not shown). The *alg-1* locus is predicted to produce two protein isoforms (Figure 2B). However, the expected molecular weights of these annotated isoforms do not fully account for the apparent difference in electrophoretic mobility of the two major isoforms detected in our Western blot experiments (Figure 2C) suggesting a possible contribution of proteolytic processing, and/or other hypothetical post-translational modifications of ALG-1. Each missense allele produces two isoforms of apparently full-length ALG-1 proteins similar to wild type (Figure 2C). The abundance of the mutant ALG-1 proteins is reduced compared to the wild type (Figure 2C), suggesting that those particular mutations may affect ALG-1 stability to various degrees. The abundance of the higher molecular weight isoform seems to be more affected by the *alg-1(anti)* alleles; however this apparent differential isoform accumulation does not appear to correlate with the severity of the *alg-1(anti)* mutant phenotypes (Table 1).

**Table 1:** *alg-1* mutations cause retarded development.

	Percentage of Young Animals with Adult Alae Synthesis^a^				
Genotype^b^	No alae	Gapped^c^	Complete^c^	(n)	Seam cell number^d^
wild-type	0	0	100	35	12.4
*lin-31(n1053)* e	0	0	100	35	12.1
*lin-31(n1053); alg-1(tm492)*	14	9	77	76	12.8
*lin-31(n1053); alg-1(ma198)*	16	3	81	32	13.0
*lin-31(n1053); alg-1(ma192)*	83	17	0	35	21.7
*lin-31(n1053); alg-1(ma195)*	27	65	8	26	20.5
*lin-31(n1053); alg-1(ma202)*	61	39	0	31	21.3
*lin-31(n1053); alg-1(ma203)*	76	24	0	29	20.6
*lin-31(n1053); alg-1(ma192)/alg-1(tm492)*	41	38	21	29	15.4
*lin-31(n1053); alg-1 (ma202)/alg-1(tm492)*	24	45	31	38	13.9

a Presence and quality of cuticular alae structures were assayed by Normarski DIC optics. Only one side of each animal was scored.

b All animals contain *maIs105* transgene which expresses an adult specific, *col-19::GFP* reporter.

c The quality of alae structure in *lin-31; alg-1(anti)* animals is extremely poor.

d Average number of seam cells per side was counted from pharynx to anus and therefore may contain 1 seam cell of the H lineage.

e *lin-31(n1053*) mutation suppresses the *alg-1(anti)* lethality by non-heterochronic mechanism.

### The novel *alg-1* mutations suppress multiple cell lineage and gene expression phenotypes of *lin-28* mutants

**Figure 3:**
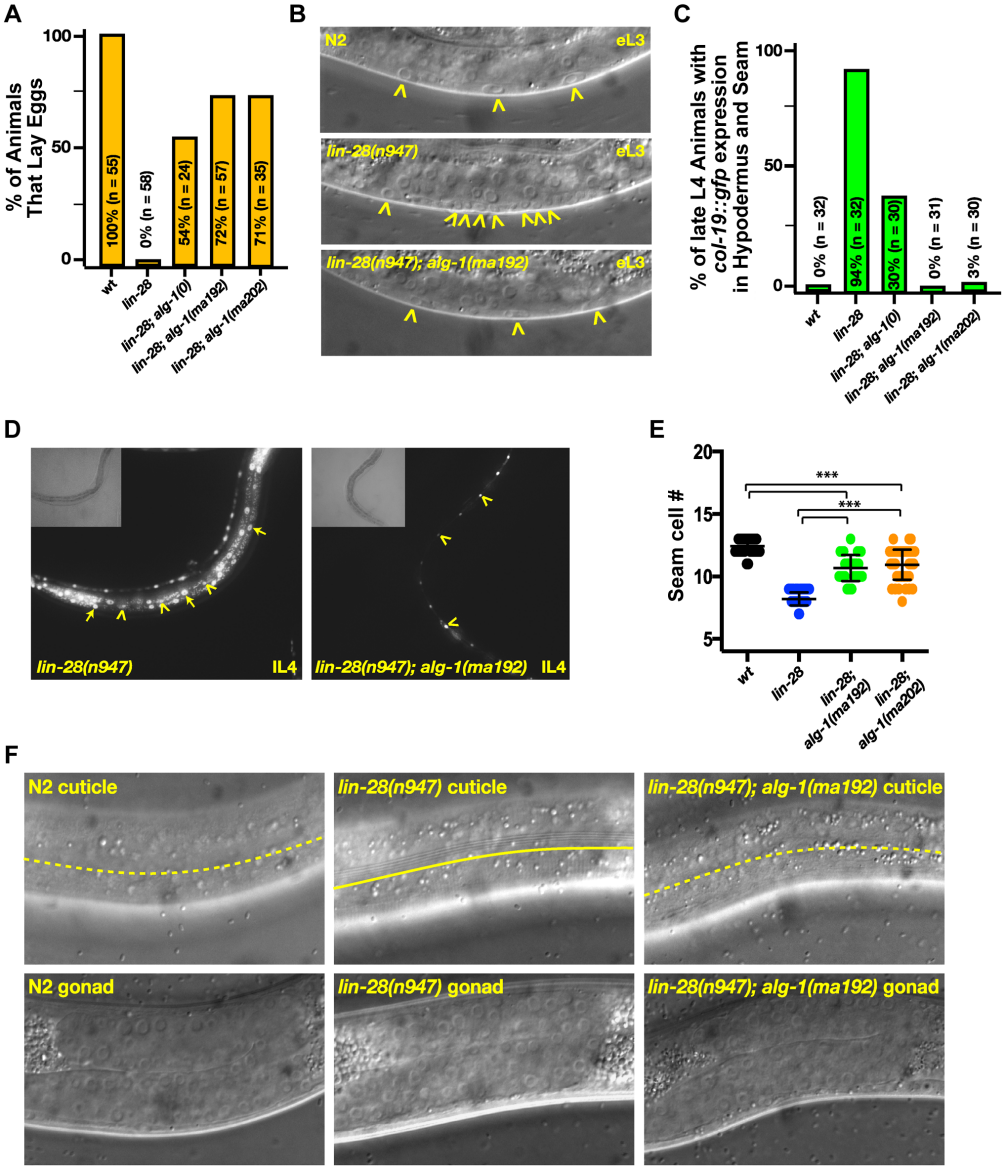
Mutations in *alg-1* suppress precocious development of *lin-28(lf)* (*lin-28(n947)*) animals. (**A**, **B**) *alg-1* mutations suppress the egg-laying defect of *lin-28(lf)* animals by suppressing the precocious divisions of the vulval precursor cells; (**B**) Early third larval (eL3) stage animals. Arrowheads indicate vulval cell nuclei. Three vulval precursor cells (P5.p, P6.p, and P7.p) are undivided in the top (N2) and bottom (*lin-28(n947); alg-1(ma192)*) panels, but in the middle panel (*lin-28(n947*), P6.p and P7.p have divided twice (one P7.p granddaughter is out of the plane of focus). (**C**, **D**) *alg-1* mutations suppress the *lin-28(lf)* precocious expression of the adult cell fate marker *col-19::gfp*. lL4-late fourth larval stage. (**E**) *alg-1* mutations also increase the seam cell number (#) produced *lin-28(lf)* mutant animals (***p<0.0001), and (**F**) suppress precocious alae formation of L4 animals; dotted line represents absence of the alae, solid line underlines the alae structure. All strains *col-19::gfp* transgene in the background. n = number of animals scored.

*lin-28(lf)* mutants skip certain developmental events characteristic of the second larval stage (L2), which results in precocious execution of cell divisions in the vulval and lateral hypodermal cell lineages. These alterations in temporal cell fates have dramatic consequences that include defects in egg laying, precocious expression of an adult-specific collagen gene, *col-19*, precocious production of adult cuticle, and precocious cessation of molting after the third (sometimes second) larval molt–one to two stages earlier than normal (Figure 3 and [18], [19]). We found that mutations in *alg-1* suppressed all of these cell-lineage and gene expression phenotypes of *lin-28(lf)* mutants (Table 2, Figure 3).

**Table 2:** *lin-28(lf)* phenotypes are suppressed by mutations in *alg-1*.

	Percentage of Animals with Adult Alae Synthesis^a^				Percentage of Animals that express *col-19::gfp* b				Average seam cell number^c^	
	L4				L4				L4	
Genotype^b^	No alae	Gapped	Comp.	(n)	No gfp	Seam only	Hyp+seam	(n)		(n)
wild-type	100	0	0	32	100	0	0	32	12.0	32
*lin-28(n947)*	0	0	100	32	0	6	94	32	8.1	32
*lin-28(n719)*	11	0	89	27	0	30	70	27	8.2	27
*lin-28(tm4566)*	20	18	62	91	2	57	41	64	8.2	64
*lin-28(n947); alg-1(ma198)*	70	20	10	30	0	70	30	30	10.6	30
*lin-28(n947); alg-1(ma192)*	100	0	0	31	0	100	0	31	10.7	31
*lin-28(n947); alg-1(ma195)*	100	0	0	32	0	100	0	32	9.7	32
*lin-28(n947); alg-1(ma202)*	97	0	3	30	0	97	3	30	10.9	49
*lin-28(n947); alg-1(ma203)*	87	10	3	31	0	100	0	31	9.5	30
*lin-28(n719); alg-1(ma203)*	64	36	0	14	7	93	0	14	10.2	13
*lin-28(tm4566); alg-1(ma202)*	68	32	0	25	24	75	0	25	9.2	17
*lin-28(n947); alg-2(ok304)*	3	9	88	34	nd	nd	nd	nd	nd	nd
*lin-28(tm4566); alg-2(ok304)*	19	14	67	37	nd	nd	nd	nd	nd	nd

a Presence and quality of cuticular alae structures were assayed by Normarski DIC optics. Only one side of each animals was scored.

b All animals contain *maIs105* transgene which expresses an adult specific, *col-19::GFP* reporter.

c Average number of seam cells per side was counted from pharynx to anus and therefore may contain 1 seam cell of the H lineage.

nd = not determined.

A striking property of the missense *alg-1* alleles isolated as suppressors of *lin-28(lf)* is that their suppression is much stronger than that exerted by *alg-1(ma198)* null allele (Table 2, Figure 3A, C). This is the case for almost all heterochronic phenotypes assessed: the premature vulval precursor cell (VPC) division and egg laying defects (Figure 3A), precocious expression of the adult-specific seam hypodermal marker, *col-19::gfp* (Table 2, Figure 3C), and precocious formation of adult-specific lateral alae on the cuticle (Table 2). These results suggest that the *alg-1* missense mutations differ in their fundamental properties from a complete loss of *alg-1*.

The *alg-1* missense mutations also efficiently suppressed the precocious seam cell division patterns of *lin-28(lf)*, thereby restoring the seam cell numbers to nearly wild type (Table 2, Figure 3E). This is noteworthy in light of previous work showing that simultaneous loss of four of seven *let-7-Family* genes (*mir-84*, *mir-48*, *mir-241*, and *let-7*) was not sufficient to suppress the reduced seam cell number of *lin-28(lf)* [20]. Therefore the potent suppression that we observe in *lin-28(lf)* carrying *alg-1* mutations suggests that the *lin-28(lf)* phenotype results from hyperactivity of microRNAs in addition to *mir-84*, *mir-48*, *mir-241*, and *let-7*. Although the newly isolated missense alleles of *alg-1* do not all appear to affect the same domain of ALG-1 (Figure 2A), all four mutants displayed similar phenotypic and genetic characteristics (Table 1, Table 2). We therefore focused our further characterizations on two of the four missense alleles, *alg-1(ma192)* and *alg-1(ma202)*.

### *alg-1* missense mutations cause heterochronic phenotypes more severe than those of *alg-1(0)* animals

**Figure 4:**
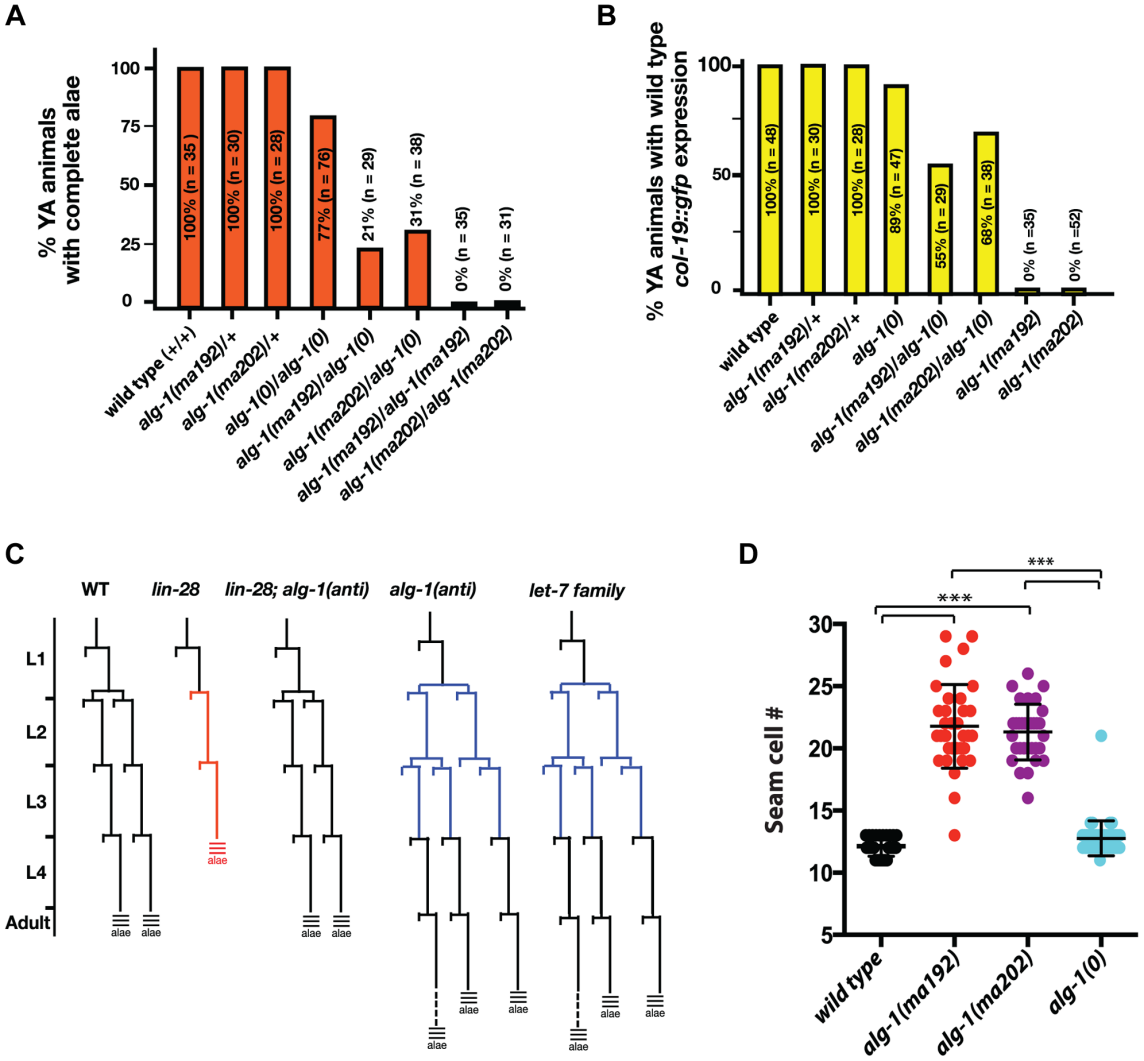
Newly isolated *alg-1* alleles are antimorphic, exhibit retarded development, and display phenotypes more severe than those of *alg-1(0)*. (**A**, **B**) Bar graphs showing the percent of young adult (YA) animals with wild type alae formation (**A**) and *col-19::gfp* adult marker expression (**B**), where *alg-1(anti)* alleles have a dosage dependent effect on both phenotypes. (**C**) Schematic of representative V1–V4 and V6 lineage cell divisions in the wild type, *alg-1(anti)*, and other heterochronic mutants. (**D**) *alg-1(anti)* mutations display increased numbers of seam cells as young adults. ***p<0.001. All strains carry *lin-31(lf)* and *col-19::gfp* in the background. The *lin-31* mutation is present in order to suppress *alg-1(anti)* vulval bursting phenotypes by non-heterochronic methods. n = number of animals scored.

To further understand the newly isolated missense alleles of *alg-1*, we phenotypically characterized the novel *alg-1* mutant animals in the absence of the *lin-28(lf)* mutation. Loss of *alg-1* is known to result in mild retarded developmental phenotypes primarily from decreased levels and/or activity of heterochronic microRNAs (Table 1, Figure 4A, B), [9]. The *alg-1(null)*(referred to as *alg-1(0)*) heterochronic phenotypes are relatively weak most likely because of the redundancy of ALG-1 with its paralog, ALG-2 and include a failure of individual lateral seam cells to terminally differentiate or to deposit an adult-specific cuticle during the larval-to-adult molt (Table 1, Figure 4A, B), [9], [32]. We found that the novel missense *alg-1* alleles also display retarded heterochronic development, but these phenotypes are much more severe than *alg-1(0)* mutants (Table 1, Figure 4A–D). Specifically, *alg-1(0)* animals display only modest retarded defects, with 77% of the young adult animals producing alae, and 89% of the young adult animals expressing a *col-19::gfp* pattern similar to the wild type (Table 1, Figure 4A, B). By contrast, *alg-1(ma202)* and *alg-1(ma192)* homozygous mutant animals completely fail to produce alae as young adults (Table 1, Figure 4A) and do not display the wild type hypodermal and seam *col-19::gfp* expression at that stage (Figure 4B). Similarly, seam cells of *alg-1(0)* animals rarely reiterate L2 cell divisions, while this phenotype is much more penetrant and expressive for the novel *alg-1* missense mutants (Table 1, Figure 4C, D). Young adult animals carrying *alg-1(ma202)* or *alg-1(ma192)* on average had 8–10 more seam cells than wild type (Table 1, Figure 4D), a phenotype similar to *let-7-Family* microRNA mutants that fail to down-regulate *hbl-1* expression [2].

### *alg-1(anti)* mutations are antimorphic

To characterize the dosage-dependence of the *alg-1(anti)* phenotypes, we examined the phenotypes of heterozygous and hemizygous *alg-1(anti)* animals. 100% of *alg-1(ma202)/+* or *alg-1(ma192)/+* animals display wild type phenotypes with respect to alae formation and *col-19::gfp* expression (Table 1, Figure 4A, B), suggesting that these alleles are either recessive or so weakly semi-dominant that we are unable to detect the dominant allelic behavior in our assays. In contrast, animals carrying one *alg-1* missense alleles in trans to an *alg-1(0)* allele are strongly retarded; only 21–31% of *alg-1(ma192)/alg-(0)* or *alg-1(ma202)/alg-1(0)* young adults produce complete alae (Figure 4A); similarly, animals of those genotypes exhibited retarded phenotypes with respect to adult-specific *col-19::gfp* expression (Figure 4B). These results suggest that one copy of *alg-1(ma202)* or *alg-1(ma192)* is more detrimental than the complete lack of ALG-1 protein. Because these missense *alg-1* mutations can cause defects more severe than those of complete loss of *alg-1*, we conclude that these mutations are antimorphs (anti) and hypothesize that they do not simply reduce ALG-1 activity, but may also interfere (directly or indirectly) with the function of the semi-redundant Argonaute ALG-2.

### *alg-1(anti)* heterochronic phenotypes involve de-repression of the *let-7-Family* microRNA target, *hbl-1*

**Figure 5:**
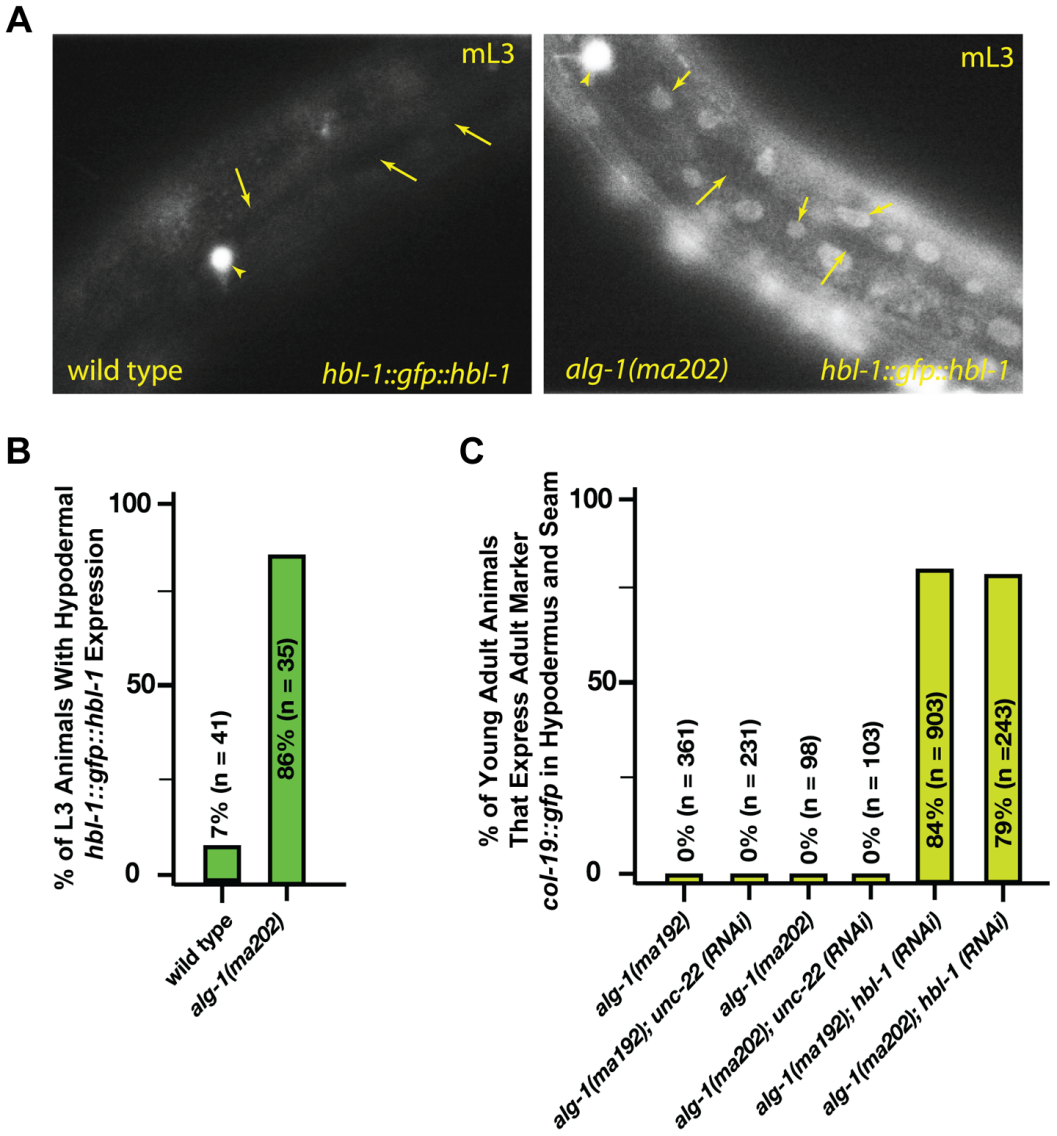
*alg-1(anti)* mutations affect *hbl-1* expression. (**A**) *hbl-1::gfp::hbl-1* 3′UTR expression in wild type and *alg-1(anti)* mutant animals. mL3-mid third larval stage. Short arrows-hypodermal nuclei; long arrows-seam cell nuclei. HSN neuron (arrowhead) is shown as a point of reference. Images were captured at identical exposure, and processed identically. (**B**) Quantification of hypodermal *hbl-1::gfp::hbl-1* 3′UTR expression in wild type and *alg-1(ma202)* animals. (**C**) *hbl-1* RNAi rescues the retarded expression of the adult marker *col-19::gfp* in *alg-1(anti)* mutant animals. All strains carry *lin-31(lf)* and *col-19::gfp* in the background. The *lin-31* mutation is present in order to suppress *alg-1(anti)* vulval bursting phenotypes by non-heterochronic methods. n = number of animals scored.

The *hbl-1* gene encodes for a transcription factor that is a direct target of the *let-7-Family* microRNAs [2], [29], [30] and is also involved in feedback regulation of *let-7* transcription [31]. Down regulation of *hbl-1* by the *let-7-Family* microRNAs is required for proper progression of cell division programs from the second to third larval stages of development (Figure 1), [2]. In wild type animals, *hbl-1* is down-regulated by *let-7-sisters* (*mir-48, mir-84, and mir-241*) between the L2 and L3 stages of larval development [2]. We observed that in the *alg-1(anti)* L3 larvae, *hbl-1::gfp::hbl-1 3′UTR* is not down regulated as it is in wild type (Figure 5A, B). Consistent with the hypothesis that increased *hbl-1* activity contributes to *alg-1(anti)* heterochronic phenotypes, knock down of *hbl-1* by RNAi in the *alg-1(anti)* background results in efficient suppression of the retarded phenotype as measured by expression of the adult-specific *col-19::gfp* reporter transgene (Figure 5C).

### *alg-1(anti)* mutations impair functions of non-heterochronic microRNAs

**Figure 6:**
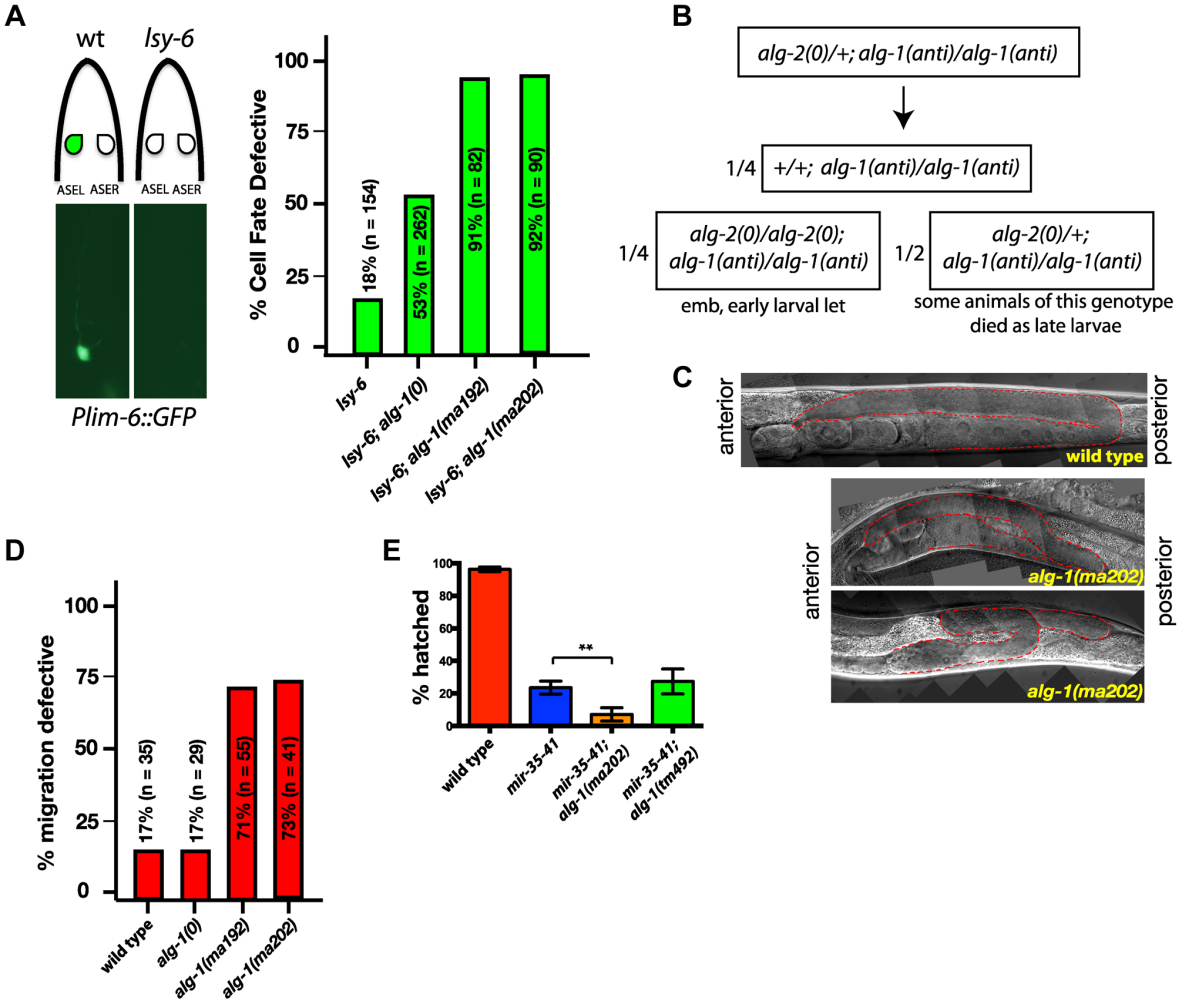
*alg-1(anti)* mutations affect functions of many microRNAs and exhibit phenotypes more severe than those of *alg-1(0)*. (**A**) *Plim-6::gfp* expression marks ASEL neuronal cell fate in wild type and mutant animals. *lsy-6(ot150)* mutants lack the *Plim-6::gfp* expression in the ASEL neurons some of the time. This phenotype is enhanced by the loss of *alg-1* and even more so in the presence of either of the two *alg-1(anti)* mutations. All strains carry *lin-31* mutation in order to suppress *alg-1(anti)* vulval bursting phenotypes by non-heterochronic methods. (**B**) Combination of *alg-1(anti)* and *alg-2(0)* mutations results in embryonic lethality (emb) and early larval lethality (let), with some late larval lethality present in *alg-2(0)/+; alg-1(anti)/alg-1(anti)* mutant animals. (**C**, **D**) Distal tip cell migration phenotypes of wild type and mutant animals. *alg-1(anti)* mutant animals display defects affecting all three phases of gonad migration. In (C) gonads are marked by a dashed line. (**E**) *alg-1(anti)*, but not *alg-1(0)* mutation enhances the embryonic lethality of *mir-35–41* mutants. (**p<0.001). All strains carry *lin-31(lf)* and *col-19::gfp* in the background. The *lin-31* mutation is present in all strains in order to suppress *alg-1(anti)* vulval bursting phenotypes by non-heterochronic methods. n = number of animals scored.

Since *alg-1(anti)* mutants display strong heterochronic phenotypes and were isolated in a screen for suppression of heterochronic phenotypes, we tested whether *alg-1(anti)* mutations affect functions of microRNAs involved in other processes. *lsy-6* is a microRNA that regulates cell fate specification of two bilaterally symmetric neurons, ASEL and ASER [36]. *lsy-6* microRNA expression in the ASEL cell down-regulates *cog-1*, the primary determinant of the ASER cell fate, thereby allowing specification of the ASEL fate [36]. Loss of *lsy-6* function results in transformation of the ASEL cell fate to that of ASER [36]. *lsy-6(ot150)* is a hypomorphic allele that alters a conserved cis-regulatory element in the *lsy-6* promoter and reduces but does not eliminate *lsy-6* microRNA function [37]. *lsy-6(ot150)* animals display an ASEL to ASER cell fate transformation of approximately 18% whereas *lsy-6(0)* animals display an essentially 100% penetrant phenotype ([37], (Figure 6A)). Using the ASEL-specific transgenic reporter, *Plim-6::gfp* [36], [38], (Figure 6A), and the *lsy-6(ot150)* sensitized background, we tested for effects of the *alg-1* mutations on the *lsy-6(ot150)* phenotype. We observed that complete removal of *alg-1* in *lsy-6(ot150)* mutant background using a homozygous *alg-1(0)* allele increases the penetrance of the ASEL to ASER fate transformation phenotype to 53% of animals (Figure 6A). However, combining the *alg-1(anti)* alleles with *lsy-6(ot150)* leads to a more dramatic enhancement, with 90% of animals displaying the ASEL to ASER transformation phenotype (Figure 6A). These data show that an *alg-1(anti)* mutation reduces the function of *lsy-6* microRNA, and does so more severely than *alg-1(0)* (Figure 6A), similar to the effect of *alg-1(anti)* on the heterochronic phenotypes.

ALG-1 and ALG-2 Argonautes function semi-redundantly, and together are essential for viability: loss of both *alg-1* and *alg-2* results in embryonic lethality [9], [39]. Similarly, combining *alg-1(anti)* with a null mutation in *alg-2* results in lethality, as determined by the absence of viable *alg-1(anti); alg-2(0)* doubly homozygous mutant animals among the progeny of heterozygous mothers (Figure 6B). *alg-1(anti); alg-2(0)* animals appeared to arrest as embryos or early stage larvae indicating that *alg-1(anti)* alleles affect functions of microRNAs normally required for viability and embryonic development [39]–[41]. In addition, we observed that *alg-1(anti)/alg-1(anti); alg-2(0)/+* animals frequently arrested as L3/L4 larvae (Figure 6B), indicating that two copies of *alg-1(anti)* can interfere with the function of a single *alg-2(+)* allele.

*alg-1(0)* mutant hermaphrodites exhibit occasional gonad migration phenotypes that can be enhanced in combination with mutations in certain microRNA genes [42], indicating that one or more microRNAs function to ensure proper gonad morphogenesis. Consistent with *alg-1(anti)* alleles affecting microRNA activity more severely than *alg-1(0)*, the distal tip cell (DTC) migration defects of *alg-1(anti)* animals were far more penetrant and more expressive than those of *alg-1(0)* (Figure 6C, D). It should be noted that the *lin-31* mutation, present in the background of all *alg-1* mutations in order to suppress *alg-1(anti)* vulval bursting, causes a mild migration phenotype on its own (Figure 6C and data not shown). Specifically, the distal tip cells of *lin-31* mutant animals migrate beyond the primary vulva site approximately 17% of the time (Figure 6D). However, *alg-1(anti)* animals display a wide range of DTC migration phenotypes during all phases of migration at a much higher penetrance than *lin-31* worms (Figure 6C, D).

Deletion of all *mir-35* family members (*mir-34–42*) results in early embryonic lethality, while deletion of *mir-35–41* (leaving *mir-42* functional) results in an incompletely penetrant temperature sensitive lethality [40], [41]. Combining the *alg-1(anti)* mutation, but not the *alg-1(0)* deletion, with the *mir-35–41* deletion enhances the *mir-35–41* temperature sensitive embryonic lethality phenotype (Figure 6E), suggesting that the *alg-1(anti)* mutations affect the functions of the *mir-35* family microRNAs in embryonic development.

### *let-7-Family* microRNA biogenesis is largely unaffected in *alg-1(anti)* mutants

**Figure 7:**
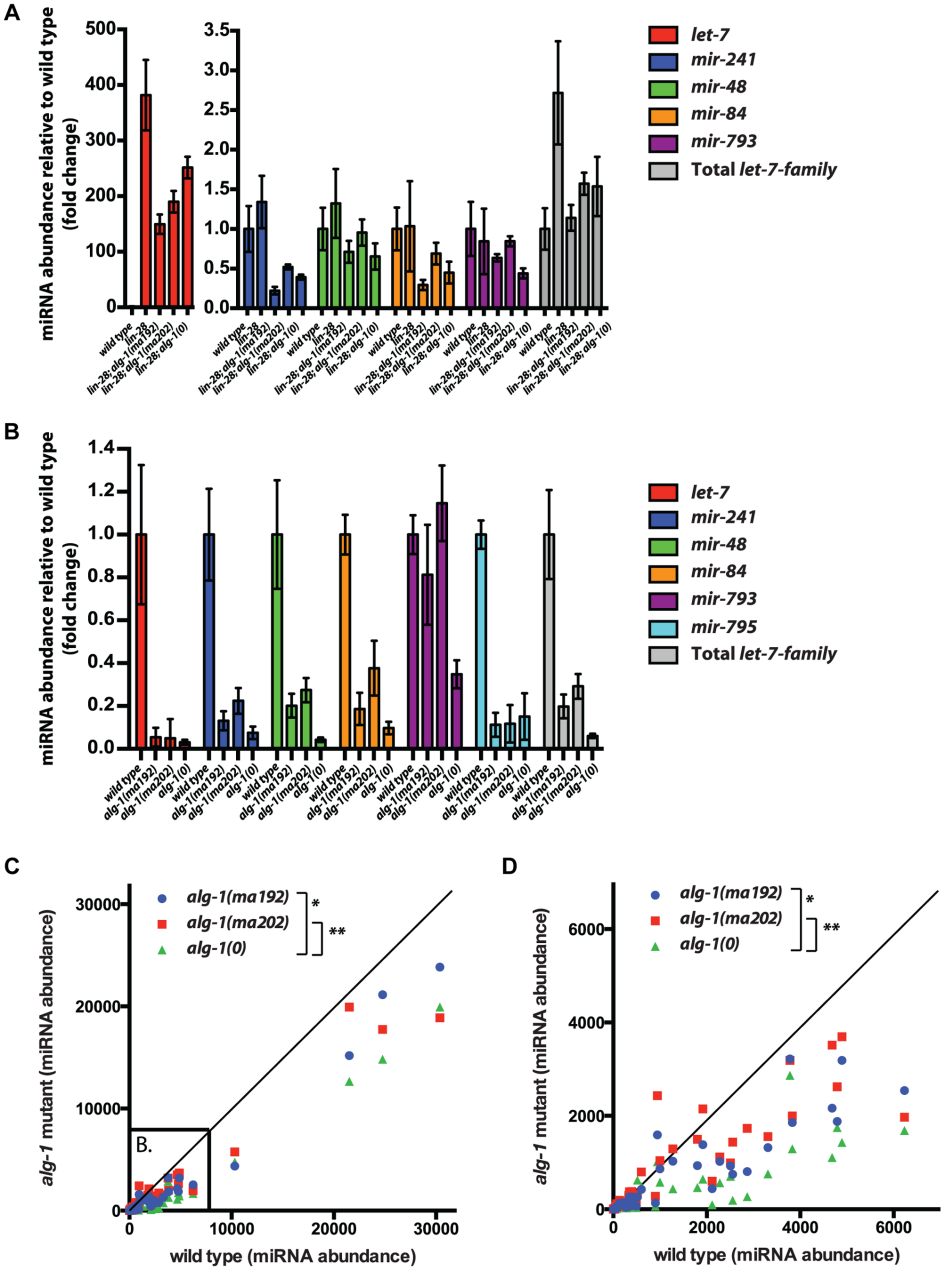
The effect of *alg-1* mutation on the levels of mature microRNAs in total RNA from L2 larvae using FirePlex miRSelect assay. (**A**, **B**) Quantification of *let-7-Family* microRNA abundance (normalized to wild type), in the context of suppression of *lin-28(0)* mutants by *alg-1* mutations (**A**), and for *alg-1* mutations in the *lin-28(+)* genetic background (**B**). Only detectable *let-7-Family* microRNAs are shown. (**A**) *let-7* microRNA levels increase dramatically in *lin-28(lf)* mutants and are reduced by the addition of the *alg-1* mutations. Levels of other *let-7* family members are not increased to statistically significant levels in *lin-28* mutants, and are either not affected or are decreased by the *alg-1* mutations to varying degrees. All strains carry the *col-19::gfp* transgene. (**B**) *let-7-Family* microRNA levels are decreased in all *alg-1* mutants compared to wild type, but *alg-1(0)* decreases microRNA abundance more than *alg-1(anti)* mutations do. (**C**, **D**) Scatterplots comparing abundance of microRNAs in total RNA from L2 larvae of wild type (X-axis, arbitrary units) and *alg-1* mutants (Y-axis, arbitrary units) using FirePlex miRSelect assays for 53 microRNAs. Complete loss of ALG-1 in *alg-1(0)* and compromised ALG-1 function in *alg-1(ma202)* and *alg-1(ma192)* results in under accumulation of microRNAs. *alg-1(ma202)* and *alg-1(ma192)* mutants have higher levels of microRNAs than *alg-1(0)* animals, *p = 0.01, **p = 0.003. (**D**) Subset of data in (C) zoomed in to show the lower abundance microRNAs. All strains in (B–D) carry *lin-31(lf)* and *col-19::gfp* in the background. The *lin-31* mutation is present in order to suppress *alg-1(anti)* vulval bursting phenotypes by non-heterochronic methods.

The suppression of *lin-28(lf)* phenotypes by *alg-1(anti)* mutations and the retarded phenotypes that *alg-1(anti)* mutants have on their own suggest that *alg-1(anti)* mutations could result in decreased levels and/or activity of the *let-7-Family* microRNAs. We therefore assayed the abundance of the let-7-Family microRNAs in RNA samples from L2 larvae (the stage where the *alg-1(anti)* heterochronic defects are induced). Vadla and colleagues previously showed that let-7 microRNA levels increase dramatically in *lin-28(lf)* animals at the L2 stage [20]. Similarly, we see a dramatic increase in let-7 microRNA levels in *lin-28(lf)* L2 larvae (Figure 7A). Interestingly, let-7 microRNA levels were decreased approximately 2-fold in *lin-28(lf); alg-1(anti)* L2 larvae compared to *lin-28(lf); alg-1(+)*, but still elevated compared to wild type levels (Figure 7A). Importantly, *alg-1(0)* and *alg-1(anti)* mutations reduced let-7-Family microRNA levels similarly in the *lin-28(lf)* background (Figure 7A). This result suggests that the more efficient suppression of the *lin-28(lf)* phenotypes by *alg-1(anti)* alleles compared to *alg-1(0)* is not entirely due to reduced let-7-Family microRNA levels, and that the ALG-1(anti) mutant proteins may be impaired in an activity or activities downstream of microRNA biogenesis. In addition, *alg-1(anti); lin-28(+)* mutants exhibit less severe reductions in let-7-Family microRNA than do *alg-1(0)* animals (Figure 7B), even though the *alg-1(anti)* heterochronic phenotypes are far more severe than those of *alg-1(0)* (Figure 4). This is also consistent with a potent effect of *alg-1(anti)* mutations on ALG-1 function(s) other than microRNA biogenesis. The absence of *lin-28* dampens the effects of *alg-1* mutations on the levels of let-7 and miR-48 microRNAs. Both let-7 and miR-48 levels are reduced at best two-fold in the absence of *lin-28* (Figure 7A), but 5–10 fold when *lin-28* is intact (Figure 7B). This would indicate that in the presence of *lin-28* these microRNAs are more vulnerable to the absence of functional ALG-1, suggesting that *alg-1* opposes the destabilizing activity of *lin-28* upon let-7 and mir-48. These data might suggest that LIN-28 may function to inhibit processing of not only let-7 (as previously shown [22]), but also miR-48.

To assess whether the relatively mild effect of *alg-1(anti)* mutations on microRNA levels (compared to *alg-1(0)*) extends to other microRNAs, we determined the levels of 53 microRNAs in wild type, *alg-1(0)*, and *alg-1(anti)* L2 larvae using the FirePlex miRSelect (Firefly) method. We found that microRNA levels were consistently and statistically significantly more reduced in *alg-1(0)* animals than in *alg-1(anti)* animals (Figure 7C, D). These data further support the idea that the more severe phenotypes of *alg-1(anti)* mutants compared to *alg-1(0)* result from impaired microRNA functions and cannot be attributed solely to reduced microRNA levels.

### Unlike *alg-1* null mutations, *alg-1(anti)* mutations do not affect pre-microRNA processing

**Figure 8:**
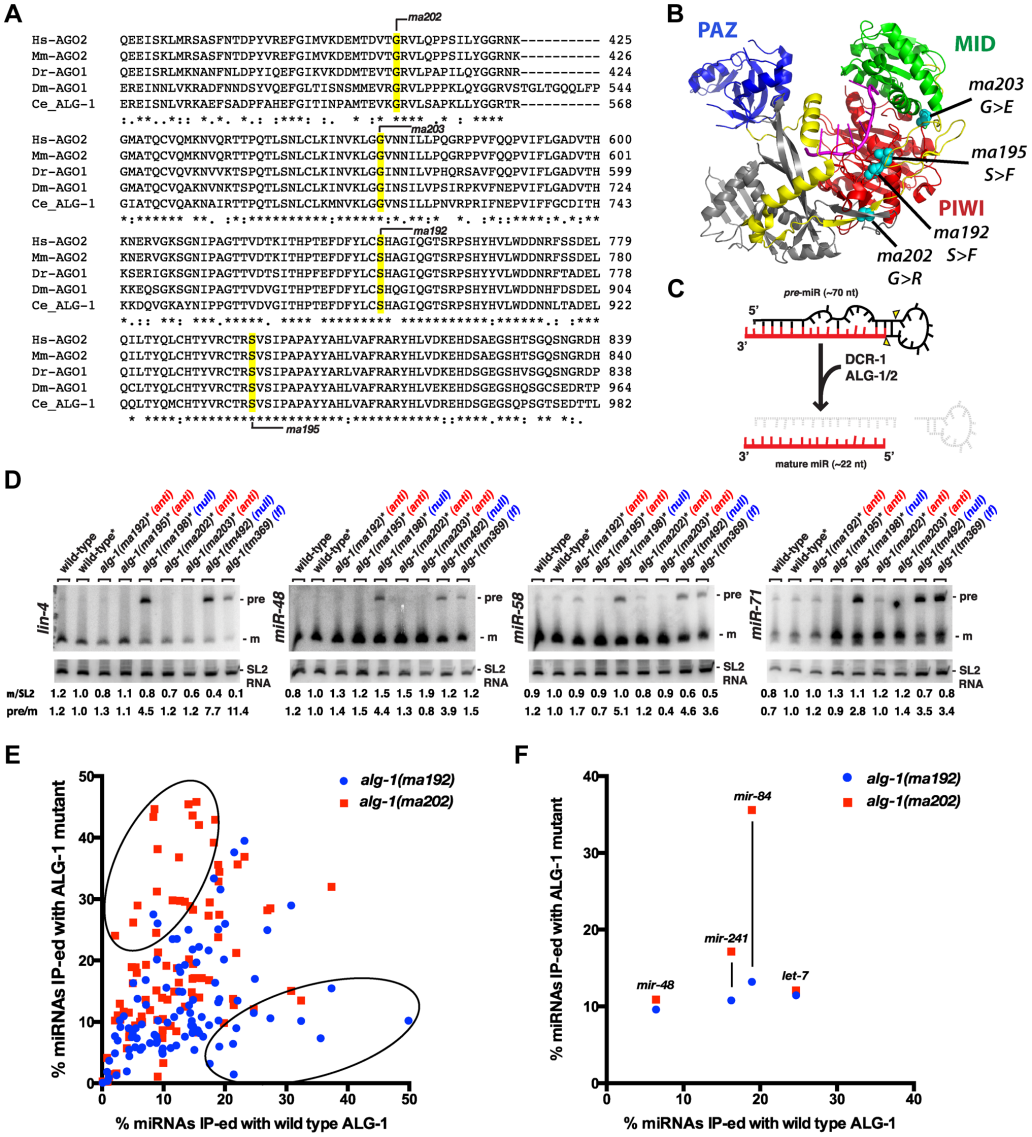
*alg-1(anti)* alleles affect conserved amino acids within the ALG-1 protein, do not affect pre-microRNA processing, and associate with microRNAs to levels comparable to wild type. (**A**) Alignments of Argonaute sequences from multiple species shows conservation of the amino acids affected by *alg-1(anti)* mutations. (**B**) Locations of conserved amino acids affected by *alg-1(anti)* mutations are mapped onto the crystal structure of hAGO2 (PDB ID 4EI1 [51]) using PYMOL. PAZ domain colored in blue, Linker 2 in yellow, MID domain in green, and PIWI domain in red. Amino acids affected by each mutation are highlighted in teal. (**C**) A schematic showing precursor microRNA processing into the mature guide microRNA. (**D**) Northern blot analysis of total RNA extracted from mixed population wild type and *alg-1* mutant animals using probes to specific microRNAs. Only *alg-1(0)* null and *alg-1(tm369)* loss of function mutants accumulate the precursor species of microRNAs. *Strains carry *lin-31, col-19::gfp* in the background. m/SL2, ratio of mature microRNA to SL2 loading control normalized to wild type *lin-31; col-19::gfp*. pre/m, ratio of precursor to mature microRNA normalized to wild type *lin-31; col-19::gfp*. (**E**) Scatterplot comparing the efficiency with which microRNAs co- immunoprecipitated wild type (X-axis) or mutant (Y-axis) ALG-1. microRNA extracted from ALG-1 immunoprecipitations was quantified using Taqman qRT-PCR. microRNA abundance in each IP was normalized to a synthetic spike-in, and also to the amount of microRNA in the starting material and to the amount of ALG-1 immunoprecipitated (IP-ed). The graph shows average % IP-ed from 3 biological replicates. (**F**) Subset of data in (E), showing % of *let-7-Family* microRNAs IP-ed with ALG-1. All strains in (E, F) carry *lin-31(lf)* and *col-19::gfp* in the background. The *lin-31* mutation is present in order to (non-heterochronically) suppress *alg-1(anti)* vulval bursting phenotypes.

The four antimorphic *alg-1* alleles affect amino acids that are evolutionarily conserved in AGO family proteins and are located in various domains of the Argonaute protein (Figure 2A, Figure 8A, B, Figure S1A). In addition, the affected amino acids are conserved in many of the non-microRNA Argonautes as well (Figure S1B). Based on published high resolution Argonaute structures [43]–[49], it appears that none of the amino acids affected by *alg-1(anti)* mutations are predicted to make direct contact with the microRNA (Figure 8A). Mapping the four amino acids affected by the *alg-1(anti)* mutations onto the crystal structure of hAGO2 [50] confirms that (Figure 8B). Interestingly, this analysis shows that the two serines affected by *alg-1(ma192)* and *alg-1(ma195)* mutations are located in the PIWI domain and directly face each other (Figure 8B).

**Figure 9:**
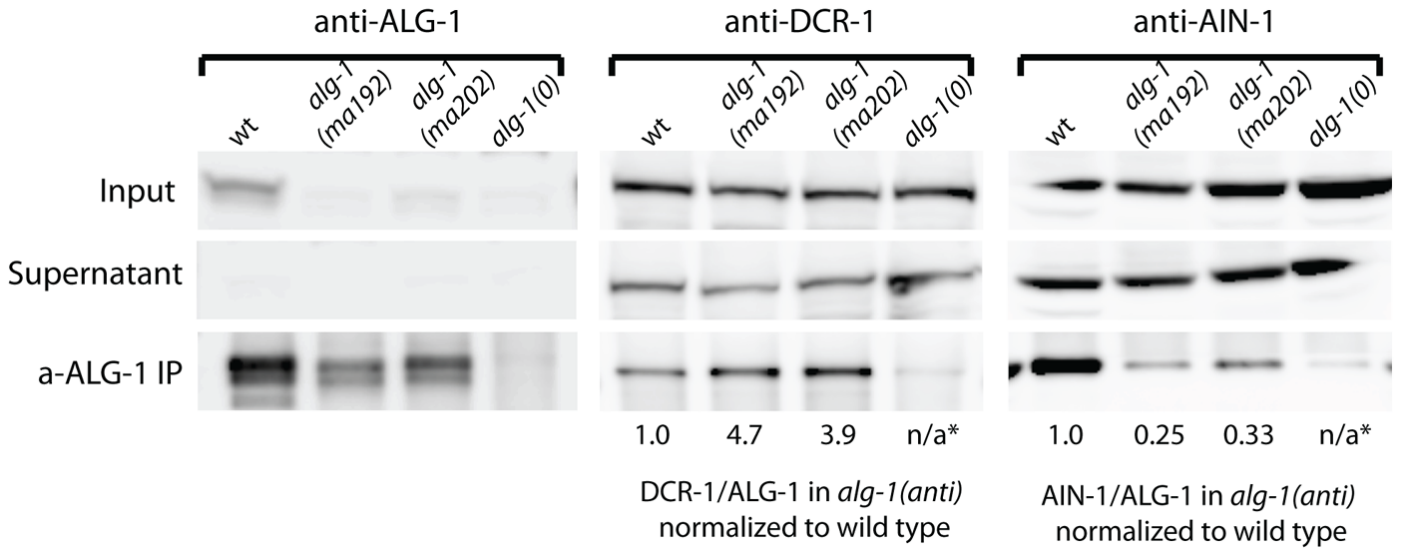
Western blot analysis of ALG-1 immunoprecipitated complexes from extracts of *alg-1(anti)* and wild type animals. Immunoprecipitated ALG-1(anti) shows an increased association with DCR-1, and a decreased association with AIN-1, compared to wild type ALG-1. The ratio of DCR-1 to ALG-1 and AIN-1 to ALG-1 were determined by quantitation of the Western blot signals, and each of those ratios for the *alg-1(anti)* mutants is normalized to that of the wild type. * means not applicable (n/a).

Mature ∼22 nt microRNAs are produced from longer precursor molecules through a series of steps involving the Dicer ribonuclease (*dcr-1*) and Argonautes *alg-1* and *alg-2* (Figure 8C), [8], [9]. Previous work showed that reduction of *alg-1* by mutation or by RNAi results in accumulation of unprocessed microRNA precursors, as is the case for reduction of *dcr-1* function [8], [9]. To determine if *alg-1(anti)* mutations affect the processing of pre-microRNAs, we performed a Northern blot analysis of total RNA from wild type, *alg-1(anti)*, and *alg-1(0)* animals (Figure 8D). The results indicate that *alg-1(anti)* animals, unlike *alg-1(0)* mutants, do not accumulate unprocessed microRNA precursors (Figure 8D). This suggests that the processing function of the ALG-1(anti) Argonautes proteins is essentially normal, and that the severe microRNA loss-of-function phenotypes of *alg-1(anti)* animals reflect defect(s) in miRISC maturation or activity downstream of microRNA precursor processing. These data also suggest that the modest reductions in microRNA levels in *alg-1(anti)* mutants (Figure 7) are not due to pre-microRNA processing defects, but rather might result from indirect effects of ALG-1(anti) proteins on microRNA stability. Interestingly, we did not observe a decrease in mature miR-48 microRNA levels by Northern blot analysis of RNA from mixed stage populations (Figure 8D), whereas Firefly assays of RNA from synchronized L2 larvae did detect a reduction of the miR-48 microRNA (Figure 7B). While there could be many reasons for this discrepancy, including the inherent differences between Northern blot and Firefly assays, it is also possible that *mir-48* may be particularly vulnerable to the loss of *alg-1* during the second larval stage.

### ALG-1(anti) mutant proteins associate with microRNAs in vivo

To determine whether microRNAs form stable complexes with ALG-1(anti) Argonaute proteins we used an anti-ALG-1 antibody to immunoprecipitate ALG-1 protein from wild type and *alg-1* mutant animals. A portion of the resulting immunoprecipitation (IP) was assessed for efficiency by Western blotting, while the remainder of the IP was used for RNA isolation. We quantified the association of 106 microRNAs with the wild type and mutant ALG-1 proteins using a multiplexed Taqman qRT assay platform. microRNA levels in each IP were normalized to: 1) a spike-in synthetic microRNA to control for differences in RNA preparation, 2) microRNA levels in the input material of the IP, and 3) the quantity of ALG-1 protein immunoprecipitated (as determined by quantitative Western blot analysis). This analysis did not indicate a pattern of overall decreased association of microRNAs with ALG-1(anti) compared to wild type ALG-1 (Figure 8E, F). The majority of the 106 microRNAs assayed fell into a class of microRNAs whose levels correlated between IP of ALG-1(anti) compared to wild type (and whose level of association changed less than two-fold between wild type and mutant). However, a subset of microRNAs appeared to be more efficiently recovered in IP of ALG-1(anti) (Figure 8E, upper oval; Figure S2A) and another subset of microRNAs appeared to be less efficiently recovered in IP of ALG-1(anti) (Figure 8E, Figure S2B). It should be noted that appreciable variability in the efficiency with which certain microRNAs were co-immunoprecipitated with ALG-1 was observed among biological replicates (Figure S2C). We do not understand the basis of this variation, but it is possible that the same, or similar causes could underlie the observed wide range of efficiencies with which various microRNAs were recovered in ALG-1(anti) immunoprecipitates. Importantly, it should be emphasized that our results do not indicated a general depletion of microRNAs in ALG-1(anti) complexes, suggesting that the functional defect of ALG-1(anti) lies downstream of the association of mature microRNA with miRISC.

### ALG-1(anti) proteins co-immunoprecipitate more DCR-1 but less AIN-1 than wild type ALG-1

To determine if ALG-1(anti) engages in stable complexes with known protein partners, we performed ALG-1 immunoprecipitations followed by Western blotting for the critical microRNA biogenesis factor DCR-1 (Dicer) and the essential miRISC effector protein AIN-1 (GW182). As expected, IP of ALG-1 from wild type animals resulted in co-precipitation of detectable quantities of DCR-1 and AIN-1 (Figure 9). In contrast, IP of ALG-1(anti) from *alg-1(anti)* animals yielded an approximately four-fold increase in the quantity of co-precipitated DCR-1 and an approximately three-fold decrease in co-precipitated AIN-1 (Figure 9). It should be noted that the ALG-1/AIN-1 ratio tended to be variable among biological replicates (not shown), although on average we observed an approximate two-fold decrease in the amount of AIN-1 co-precipitated with ALG-1(anti), compared with the wild type. Perhaps ALG-1(anti)/AIN-1 complexes are relatively unstable under the conditions of our IP experiments, which could render the yield of co-precipitation of AIN-1 with ALG-1(anti) particularly sensitive to experimental perturbations. These data are consistent with the hypothesis that the functional defect of ALG-1(anti) containing miRISC is at a step subsequent to Dicer processing of pre-miRs and microRNA loading, and prior to transition of miRISC to effector activity.

### ALG-1(anti) proteins interact with RNA duplexes *in vitro* and retain target cleavage activity similar to wild type ALG-1

**Figure 10:**
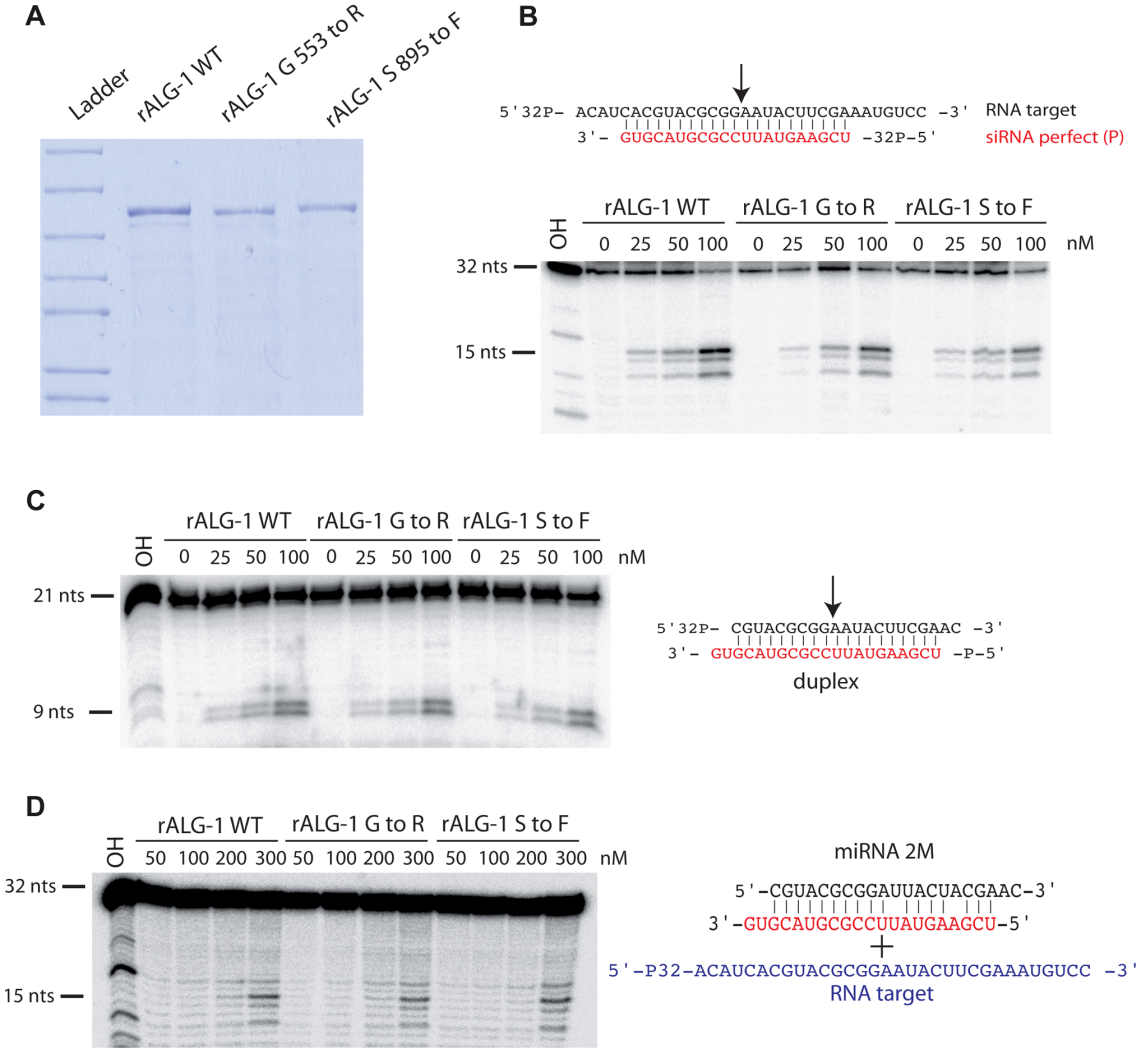
Purified recombinant ALG-1(anti) retain the slicing ability similar to the wild type ALG-1. (**A**) SDS-PAGE gel of recombinant ALG-1 proteins. Wild type ALG-1, ALG-1 G553R and ALG-1 S895F were bacterially expressed and purified. (**B**) Radiolabeled target RNA is cleaved by rALG-1 proteins preloaded with a siRNA (red) to produce a major 15 nt cleavage species as well as two minor cleavage species. (**C**) rALG-1 proteins, including rALG-1(anti), bind and cleave a perfectly base-paired duplex. (**D**) rALG-1 proteins bind a microRNA duplex containing containing two mismatches. Pre-bound rALG-1/duplex complexes are able to cleave an RNA target, producing a 15-nt major cleavage RNA species.

ALG-1 has been previously shown to possess slicing activity *in vitro*, an activity which contributes to miRISC maturation [8]. We examined if the ALG-1(anti) proteins retained their ability to cleave an RNA target *in vitro*. Proteins encoded by both *alg-1(ma192)* and *alg-1(ma202)* alleles were GST-tagged, expressed in *E. coli*, and purified to obtain rALG-1(S895F) and rALG-1(G553R), respectively (Figure 10A). The ability of each rALG-1 protein to cleave a perfect target was assessed by pre-loading each protein with a single stranded siRNA and then subsequently presenting a perfectly complementary target (Figure 10B). Both rALG-1(S895F) and rALG-1(G553R) were able to cleave a target at an efficiency similar to wild type rALG-1 (Figure 10B). We next tested whether rALG-1(S895F) and rALG-1(G553R) proteins could cleave the passenger strand of an siRNA duplex (Figure 10C). We found that rALG-1(S895F) and rALG-1(G553R) were able to both load the duplex and cleave the passenger-like strand of the duplex (Figure 10C). Finally, we took advantage of the *in vitro* assay cleavage to indirectly assess the ability of the ALG-1(anti) to interact with the mRNA targets. rALG-1, rALG-1(S895F), and rALG-1(G553R) were pre-incubated with a microRNA-like duplex containing two mismatches, and then challenged with an RNA target (Figure 10D). Both mutant rALG-1 proteins were able to induce cleavage of the target RNA with efficiency similar to wild type (Figure 10D), suggesting that both rALG-1(S895F) and rALG-1(G553R) could release the passenger strand of the duplex microRNA in order to interact with the target, at least under the *in vitro* conditions of this assay. While the cleavage reactions were found to be inefficient under the *in vitro* conditions of the assay (possibly due to the fact that Argonaute proteins can purify with small RNA already occupying the protein, [50], [51]), the data nonetheless suggest that the rALG-1(anti) proteins retain their catalytic activity to levels similar to wild type.

The results that bacterially expressed ALG-1(anti) proteins can be catalytically active, taken together with our findings that ALG-1(anti) proteins support Dicer processing and associate with mature microRNAs in worms, lend strong support to a model wherein the ALG-1(anti) proteins are defective in a previously-unrecognized, post-processing Argonaute miRISC maturation activity.

## Discussion

### Novel Argonaute mutations globally disrupt microRNA activity

Here we report the identification of point mutations in conserved amino acids of a microRNA Argonaute ALG-1 that confer novel and informative biochemical and genetic properties. These mutations cause stronger phenotypes than *alg-1* null alleles, and hence are referred to as “antimorphic” (“anti”). Normally, the two orthologous proteins ALG-1 and ALG-2 exhibit nearly redundant function, such that genetic knockout of one ortholog causes only weak or undetectable phenotypes [9], [39]. By contrast, the *alg-1(anti)* mutations we describe here cause severe developmental defects consistent with dramatic impairment of *let-7-Family* microRNA activity, impair functions of other microRNAs including *lsy-6* and *mir-35-Family*, and result in lethality in combination with *alg-2(0)*, consistent with broad defects in the activity of additional microRNAs. We interpret these results to indicate that the *alg-1(anti)* mutations not only impair ALG-1 function, but they also cause the mutant ALG-1 protein to (directly or indirectly) compete with another component of miRISC, presumably the semi-redundant ALG-2. Our observation that the *alg-1(anti)* alleles are recessive to *alg-1(+)* suggests that the mutant ALG-1(anti) proteins may be more effective in competing with wild type ALG-2 than they are with wild type ALG-1.

How might ALG-1(anti) proteins interfere with ALG-2 activity? Although it is possible that the mutant ALG-1(anti) may inhibit ALG-2 by direct ALG-1/ALG-2 physical interaction, Argonaute proteins are not known to form heterodimers. It is also possible that *alg-1(anti)* may affect regulation of *alg-2* by decreasing the amount of ALG-2 protein produced. However, we found that *alg-1(anti)* mutations did not significantly reduce the amount of *alg-2* mRNA (Figure S3). While it is possible that ALG-2 protein accumulation may still be affected by the *alg-1(anti)* mutations, such effects would not appear to be via regulation of the *alg-2* transcript.

**Figure 11:**
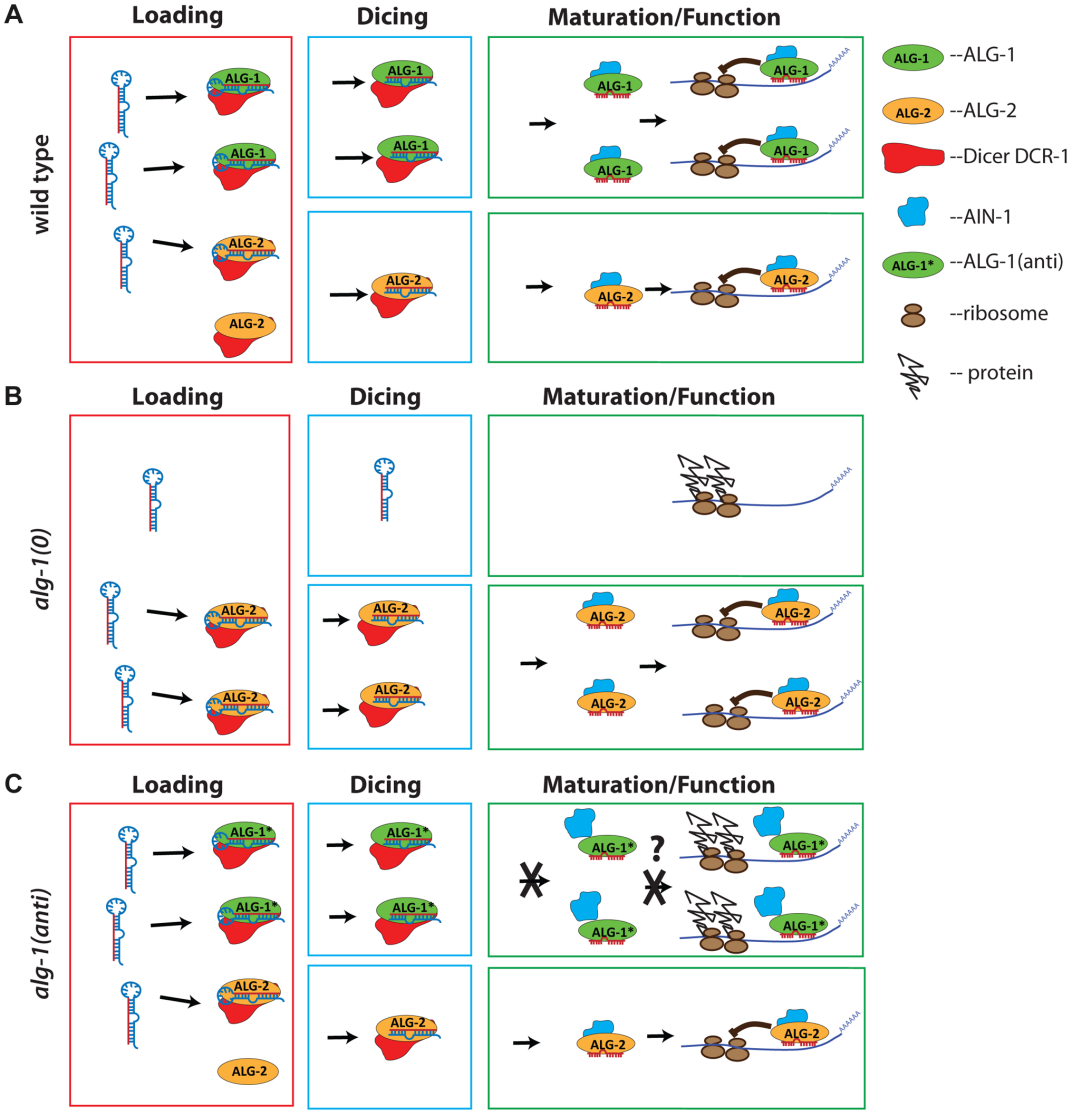
Model representing a proposed mode of action of antimorphic mutant ALG-1. (**A**) In wild type animals, ALG-1 and ALG-2 associate with microRNA precursors (Loading), act in conjunction with Dicer to facilitate cleavage of the precursor to yield mature microRNA (Dicing), and through a series of miRISC maturation steps, associate with effector partners including AIN-1/GW182, and repress mRNA targets (Maturation/Function). According to the model, ALG-1 and ALG-2 are partially redundant, and function asymmetrically; ALG-1 carries a majority share of microRNA function. (**B**) In the absence of ALG-1, ALG-2 is able to partially cover for reduced overall ALG activity, and so *alg-1(0)* animals exhibit weak microRNA loss-of-function phenotypes, and accumulate abnormal levels of microRNA precursors. (**C**) In *alg-1(anti)* animals, microRNA activity is globally poisoned by the ALG-1(anti) protein. This is because ALG-1(anti) protein is competent for Loading and Dicing, but is blocked in one or more steps of miRISC maturation wherein ALG-1 would normally transition from Dicer associated microRNA processing to AIN-1/GW182 associated target repression. This hypothetical miRISC maturation defect results in sequestration of miRISC components (including microRNAs) in inactive complexes. Jagged lines attached to ribosomes (brown ovals) represent un-repressed protein synthesis from target mRNA.

We propose that ALG-1(anti) mutant proteins essentially compete with ALG-2 in trans by sequestering microRNAs and possibly other limiting miRISC components (such as Dicer) into nonfunctional complexes (Figure 11). While *alg-1(anti)* mutants show reduced amounts of ALG-1 protein, our IP experiments show that ALG-1(anti) protein associates with microRNAs to levels similar to wild type ALG-1, when normalized to the amount of immunoprecipitated ALG-1. Therefore, unlike genetic contexts where *alg-1* is deleted and all the microRNAs are free to associate with ALG-2 (Figure 11B), the presence of ALG-1(anti) protein may result in some of the microRNAs being partitioned into defective ALG-1(anti) complexes and sequestered from ALG-2 (Figure 11C).

What could be defective about ALG-1(anti) miRISC? Our *in vitro* biochemical analysis of ALG-1(anti) proteins indicates that the mutant Argonautes can load a guide RNA and are capable (at least under our assay conditions) of eliciting slicer activity to a level that is indistinguishable from wild type ALG-1. It was previously shown that ALG-1 slicer activity is important for function *in vivo*, likely due to passenger strand cleavage [8]. In addition, the ability of ALG-1(anti) proteins to bind microRNAs suggests that ALG-1(anti) mutants are likely defective in other step(s) of miRISC maturation, turnover and/or effector phases of microRNA-mediated repression. The *alg-1(anti)* mutations could affect the structure of the ALG-1 protein, and/or affect essential protein-protein interactions. Indeed, we find that a substantially larger fraction of ALG-1 population associates with DCR-1 in *alg-1(anti)* mutants. These findings, considered together with our observation of a reduced association of ALG-1(anti) with the miRISC effector protein AIN-1, are consistent with the model that ALG-1(anti) miRISC is impaired in maturation from biogenesis to effector activity, thereby sequestering a critical fraction of microRNAs and perhaps other essential miRISC components in nonfunctional complexes (Figure 11). It should be noted that it might not be necessary for this hypothetical microRNA sequestration to be quantitative in order to reduce the amounts of functional miRISC below phenotypic thresholds.

According to this model it is also possible that titration of DCR-1 activity could occur in *alg-1(anti)* animals, since DCR-1 associated with ALG-1(anti) complexes would presumably be unavailable for processing of additional microRNA precursors. In that event, we might have expected to observe precursor accumulation, and a corresponding reduction of mature microRNA levels. While our data do show a modest reduction in mature microRNA levels in *alg-1(anti)* mutants, precursor accumulation was not detectable, at least not within the sensitivity of our Northern blot assay. This suggests that the fraction of DCR-1 associated with ALG-1(anti) may not be sufficient to significantly deplete the pool of DCR-1 available for precursor processing.

### ALG-1 microRNA biogenesis and effector functions

*alg-1(0)* mutants accumulate unprocessed microRNA precursor hairpins, similar to Dicer (*dcr-1*) loss of function [8], [9] (see also Figure 8), suggesting that ALG-1 directly or indirectly functions as a cofactor for Dicer cleavage of pre-microRNA hairpins. This implies that ALG-1 is a multifunctional protein with roles in microRNA biogenesis as well as in miRISC effector activity. A role for ALG-1 as a functional co-factor that supports the pre-microRNA processing by Dicer is also supported by both co-IP and proteomic data [52], [53]. Moreover, properties of the *alg-1(anti)* mutations described here also support a multifunctional role for ALG-1 in the microRNA pathway. Two striking properties of *alg-1(anti)* mutations are that ALG-1(anti) proteins appear to associate with mature microRNAs to normal levels, and moreover, unlike *alg-1(0)* or *alg-1(lf)* animals, *alg-1(anti)* mutants do not accumulate unprocessed microRNA precursors. Thus, the ALG-1(anti) mutant proteins seem to retain the capacity to promote Dicer cleavage of microRNA precursors and to associate with mature microRNAs. However, our observation that ALG-1(anti) co-immunoprecipitates with more DCR-1 and less AIN-1 than wild type ALG-1 suggests that ALG-1(anti) may be impaired in a maturation process involving DCR-1 release and AIN-1 association. It should be noted that, although all four existing *alg-1(anti)* alleles display similar phenotypes, they may not necessarily affect precisely the same step(s) of miRISC maturation downstream of microRNA biogenesis and Argonaute loading.

### *alg-1(anti)* developmental timing phenotypes

We recovered mutations affecting general microRNA function as suppressors of the developmental timing mutant, *lin-28(lf)*. This indicates that *lin-28(lf)* phenotypes for the most part involve hyperactivity of microRNAs. What microRNA(s) in particular? Since *lin-28(lf)* mutants over-express the *let-7* microRNA in the L2 stage (Figure 7A and [21]), *let-7* (perhaps in combination with other *let-7-Famil*y microRNAs) was a plausible candidate. However, previous findings showed that the precocious L3 fate phenotype of *lin-28(lf)* larvae cannot be entirely attributed to *let-7-Family* hyperactivity: mutations that simultaneously remove three (*mir-48, mir-84, mir-241*), or even all four (*mir-48, mir-84, mir-241, let-7*) of the major *let-7-Family* microRNAs did not suppress the precocious L3 fates of *lin-28(lf)* [2], [20]. These data, in combination with our observation that *lin-28(lf)* seam cell lineage defects are suppressed by *alg-1(anti)* are consistent with the idea that other microRNAs in addition to *mir-48, mir-84, mir-241*, and *let-7* may be hyperactive in *lin-28(lf)* and may contribute to controlling the L2-L3 transition.

The particular *alg-1(anti)* mutations identified here were isolated as viable suppressors of *lin-28(lf)*. More severely antimorphic ALG-1 mutant proteins that more effectively compete with ALG-2 might have resulted in lethality, and hence would not have been recovered in our *lin-28(lf)* suppressor screen. Nevertheless, this screen did yield mutations that generally affect microRNA functions and therefore supports the possibility that further screening may lead to isolation of animals carrying mutations in other miRISC components, provided such mutations do not result in lethality. An *alg-1(he210)* missense mutation was also previously isolated in a screen for disorganized seam cells [54]. The strength of the phenotypes reported [54], as well as the occurrence of similar seam cell disorganization phenotypes in our antimorphic mutants (data not shown) is consistent with the *alg-1(he210)* allele also being antimorphic in nature.

### Genetic dissection of Argonautes

Redundant genes can be notoriously resistant to genetic analysis by straightforward loss-of-function genetics, since all or most of the set of redundant genes may need to be simultaneously eliminated to cause a phenotype. However, our results illustrate how antimorphic mutations that partially disable one member of a redundant family of proteins can poison the collective functions of the family, and thereby produce phenotypes that reveal activities not otherwise accessible by conventional forward genetic screens. In principle, different antimorphic alleles could affect different aspects of ALG-1 function. Therefore the genetic suppressor screen used here to recover *alg-1(anti)* mutations offers the potential for genetic dissection of Argonaute functionality.

ALG-1 and ALG-2 proteins are 77% identical, and ALG-2 contains the same conserved residues as those affected in the *alg-1(anti)* [39]. The genetic redundancy and conservation of molecular function raises interesting questions including: why did we recover antimorphic alleles of *alg-1* but not *alg-2*? We do not believe that the screen was saturated, and it is possible that further screening may result in recovery of hypothetical *alg-2(anti)* mutations. Alternatively, perhaps ALG-1 and ALG-2 are not entirely equivalent functionally or biochemically, such that ALG-2 antimorphic mutations might be either too potent (and hence lethal) or too weak to be recovered in our screen. There is evidence that ALG-1 and ALG-2 are not strictly functionally equivalent. We observed that loss of *alg-1* can partially suppress *lin-28(lf)* mutant phenotypes, but loss of *alg-2* Argonaute does not (Table 1). Loss of *alg-1* results in overt, albeit mild, heterochronic developmental abnormalities, while *alg-2(0)* mutants display no detectable heterochronic phenotypes (Table 2 and data not shown). This suggests that ALG-1 plays a greater role in heterochronic microRNA function than does ALG-2. Other distinctions have been reported between ALG-1 and ALG-2 in their expression patterns and profiles of associated microRNAs [39], and in their relative roles in microRNA processing [8]. Other genetic evidence also points to additional functional non-redundancy between ALG-1 and ALG-2 [55].

Our data indicate that the *alg-1(anti)* mutations are synthetic lethal in combination with *alg-2(0)*, which suggests that recovery of viable antimorphic mutations in an Argonaute could only occur in animals where there are multiple microRNA Argonaute genes, as is the case for *C. elegans*, or mammals, where there are four Argonaute family proteins that function with microRNAs [56]–[58]. The nematode *alg-1(anti)* mutations described here are all substitutions in amino acids that are broadly conserved in Argonaute family proteins, including the four mammalian miRISC Argonautes, AGO1 – AGO4 (Figure S1). It remains to be determined if the detrimental effects of the *alg-1(anti)* mutations are a peculiarity of *C. elegans* or whether the corresponding amino acid substitutions in other Argonautes-in *C. elegans* or in other organisms-can cause antimorphic effects on microRNA activity. Such hypothetical antimorphic mutations in mammalian Argonautes could contribute to diseases that involve microRNAs, such as cancer. For example, global reduction of microRNA activity has been shown to be oncogenic in certain circumstances [59]. Wilms tumors often carry a deletion of three AGO genes [60], [61], and mutations of AGO2 were frequently found in colorectal and gastric cancers [62]. In addition, Dicer1 mutations are associated with many tumors (reviewed in [63]), and *Dicer1* functions as a haploinsufficient tumor suppressor [64], and so mammalian *Ago(anti)* mutations could predispose cells to tumor formation.

## Methods

### Strains and *C. elegans* culture

*C. elegans* culture was performed using standard nematode growth conditions [18], [19], [65]. All strains were grown at 20°C unless otherwise noted. To obtain synchronized nematode populations, gravid adults were treated with hypochlorite, the resulting eggs were hatched overnight in M9 and then placed onto *E. coli* lawns for recovery and growth. Animals were collected as young L2 larvae following the first-molt lethargus. Data shown for *alg-1* null (*alg-1(0)*) was generated using the *alg-1(tm492)*, except in Figure 7, where *alg-1(ma198)* null allele was used. Because of their strong heterochronic phenotypes, *alg-1(anti)* mutant animals often burst through the vulva during the L4-adult molt. Therefore *alg-1(anti)* mutations were maintained in a *lin-31(n1053)* genetic background that impairs vulva development and thereby suppresses the bursting phenotype of *alg-1* mutants while leaving their heterochronic phenotypes intact. The *col-19::gfp* adult marker is also present in all the strains unless otherwise noted. Therefore, unless otherwise noted, all strains contain *lin-31; col-19::gfp* and their phenotypes are compared to their wild type equivalent *lin-31; col-19::gfp*.

### Screen for *lin-28* suppressors; mutation mapping and cloning

*lin-28(n947)* animals were mutagenized for four hours using 0.05 M ethylmethylsulfonate (EMS), and their F2 progeny were screened for suppression of the severe morphological and egg-laying defects characteristic of *lin-28(lf)* mutants. Suppressor mutations were backcrossed to CB4856 Hawaii strain and mapped using the snip-SNP protocol as previously described [66]. The region containing the mutation was scanned for candidate genes, which were subsequently sequenced.

### Phenotypic characterizations

Heterochronic phenotypes were assessed by scoring for the stage-specificity of adult lateral alae on the cuticle surface and the expression of an adult-specific hypodermal reporter transgene *col-19::gfp* [19], [67]. Differential interference (DIC) microscopy was used to score seam cell number in L4 stage larvae or young adult animals, the presence of lateral alae on L4 or young adult animals, and gonad morphology in young adult stage animals. The *col-19::gfp* expression was scored using a fluorescence equipped dissecting or a compound microscope. ASEL/ASER fates were determined by assessing the *Plim-6::gfp* transgene expression that is specific to ASEL in the wild type. Expression of the *Plim-6::gfp* reporter was observed using a fluorescence equipped Zeiss dissecting scope. Enhancement of *mir-35–41* embryonic lethality phenotype was assessed by determining the percentage of embryos capable of hatching for *mir-35–41* embryos in combination with *alg-1* mutations (the *lin-31* mutation was present in the background of all strains to keep the *alg-1* animals from bursting through the vulva). L4 animals were placed at 25°C for 24 hours, after which the animals were cut open to release the eggs. The eggs were placed at 25°C and assessed for their ability to hatch following a 16 hours incubation, and reassessed 12 hours later. It should be noted that many animals with the *mir-35–41* deletion arrested as larvae after hatching.

### microRNA quantification by FirePlex miRSelect

Total RNA was extracted from worms using Trizol (Invitrogen). For samples yielding greater or equal to 1 micrograms of total RNA, microRNA abundance was determined by using Firefly Bioworks FirePlex miRSelect method [68] with a custom miRSelect panel using standard kit protocol. In short, RNA samples were hybridized to the custom microRNA-specific probes coated on the FirePlex hydrogel beads. A universal biotinylated adapter was then ligated onto the captured microRNAs and labeled with a fluorescent reporter. The level of fluorescence for a given particle (and therefore a given microRNA) was detected using a Guava easyCyte 8HT flow cytometer. Assays for U18 RNA were included as normalization controls for RNA input. Data was analyzed using FireCode software (Firefly Bioworks), and microRNA abundance was normalized to U18 RNA levels.

### Northern blot analysis

Total RNA was isolated from a mixed population of animals, and Northern blots were performed as previously described [69], [70]. Density of the signal was quantified using ImageJ software.

### RNAi

*hbl-1(RNAi)*, *unc-22(RNAi)*, and empty vector control (L4440) RNAi was performed by feeding the worms *E. coli* producing dsRNA as described [71]. Animals were fed RNAi food starting as starved L1s, and throughout development. Phenotypes were scored when the animals reached the young adult stage.

### Immunoprecipitations and western blot analysis

To assess ALG-1 protein levels, total protein was extracted from mixed stage animals as previously described [72], except RNAse inhibitor (Invitrogen) was added to the lysis buffer. Western blotting was performed as previously described [72]. ALG-1 custom polyclonal rabbit antibody (Anaspec) was used for ALG-1 protein detection as described [72]. ALG-1 immunoprecipitations were performed as previously described using a custom ALG-1 specific polyclonal antibody (Anaspec) [72]. In short, whole protein lysis sample was divided into two equal parts: one was used for subsequent ALG-1 immunoprecipitation(IP), one for RNA preparation in order to determine the amount of microRNAs present in the starting material. Half of the immunoprecipitated sample was used to assess the efficiency of immunoprecipitation by western blotting for ALG-1 protein, and the other half was used for RNA preparation. For protein co-IP, the precipitated material was probed with a custom rabbit polyclonal ALG-1 antibody [69], with a rabbit anti-DCR-1 antibody [52], or a rat anti-AIN-1 antibody [11].

### microRNA quantification by qRT-PCR (Taqman)

Total RNA was extracted from worms using Trizol (Invitrogen). For samples with total RNA yields less than 1 microgram (such as ALG-1 associated RNAs recovered by immunoprecipitation) and the corresponding starting material samples, microRNA levels were determined using a panel of Taqman-based real time PCR assays (Applied Biosystems). RNA was extracted in the presence of a synthetic *Arabidopsis mir-159* spike-in; all Ct values were first normalized to the synthetic *mir-159* spike-in in order to control for variation in RNA sample preparation. Ct values resulting from the qRT-PCR performed on the immunoprecipitated RNA were also normalized to the amount of ALG-1 protein recovered by the immunoprecipitation. The Ct values resulting from the qRT-PCR performed on the input RNA were normalized to the mass of starting RNA material. Percent of microRNA immunoprecipitated with ALG-1 was determined from the ratio of microRNA in the IP sample to the starting material sample.

### Recombinant protein purification and rALG-1/RNA binding and slicing assays

Recombinant *C. elegans* ALG-1 protein purification and ALG-1/RNA binding and slicing assays were performed as previously described [8].

## Supporting Information

Figure S1 (**A**) Alignments of human AGO1-4 shows conservation of the amino acids affected by *alg-1(anti)* mutations. (**B**) A phylogenetic tree of *C. elegans* AGO proteins. Colored squares next to the AGO genes represent the presence of the amino acids affected by the *alg-1(anti)* mutations. Tree was created with Geneious using the nearest neighbor method.
(TIF)

Figure S2 (**A**) A subset of microRNAs (highlighted by the oval in the top left quadrant of Figure 8E) that on average showed increased immunoprecipitation with ALG-1(anti) versus wild type ALG- and were immunoprecipitated at efficiencies of approximately 20% or more. Each dot represents a microRNA from a single biological replicate, all three replicates are plotted to show variability. (**B**) A subset of microRNAs (highlighted by the oval in the bottom right quadrant of Figure 8E) that on average showed a decreased association with ALG-1(anti) versus wild type ALG-1 and were immunoprecipitated at efficiencies of approximately 20% or more. Each dot represents a microRNA from a single biological replicate; all three replicates are plotted to show variability among replicates. (**C**) Scatterplot comparing microRNA association with wild type ALG-1 protein in three biological replicates. Technical variation between replicates results in imperfect correlation between the data sets. RNA isolated from ALG-1 immunoprecipitations was quantified using Taqman qRT-PCR. microRNA abundance in each IP was normalized to synthetic spike-in and to the amount of microRNA in the starting material. Data are plotted as % of microRNA levels in the starting material that had immunoprecipitated (IP-ed) with ALG-1. (**D**) Scatterplot comparing microRNA association with immunoprecipitated wild type (X-axis) and mutant (Y-axis) ALG-1. RNA isolated from ALG-1 immunoprecipitations was quantified using Taqman qRT-PCR. microRNA abundance in each IP was normalized to a synthetic spike-in and the amount of microRNA in the starting material, but not the amount of ALG-1 immunoprecipitated (IP-ed). Data are plotted as % of microRNA in the starting material that had IP-ed with wild type or mutant ALG-1. The graph shows average % IP-ed from 3 biological replicates. All strains carry *lin-31(lf)* and *col-19::gfp* in the background. *lin-31* mutation is present in order to suppress *alg-1(anti)* vulval bursting phenotypes by non-heterochronic methods.
(TIF)

Figure S3 (**A**) A schematic showing positions of the two sets of RT-PCR primers within the *alg-2* transcript. Only exons 4, 5, and 6 are drawn, with dash line representing the rest of the molecule. (**B**) Effects of *alg-1* mutations on levels of *alg-2* RNA as determined by qRT-PCR. Levels of *alg-2* RNA are reduced approximately 2-fold in the *alg-(0)* mutants as compared to wild type, and less than 2-fold in *alg-1(anti)* mutants as compared to wild type. qRT-PCR was performed on RNA prepped from 3 biological replicates of *alg-2(ok304)* mixed stage animals using the Qiagen Quanti-fast kit. The results were normalized to tubulin RNA levels, and the ratio of *alg-2* levels was calculated using the ΔΔCt method (Applied Biosystems).
(TIF)
