# Supplementary figures and images for: Mutations in Conserved Residues of the C. elegans microRNA Argonaute ALG-1 Identify Separable Functions in ALG-1 miRISC Loading and Target Repression
Source: PLoS Genet. 2014 Apr 24;10(4):e1004286. doi: 10.1371/journal.pgen.1004286 (PMC3998888; doi:10.1371/journal.pgen.1004286)

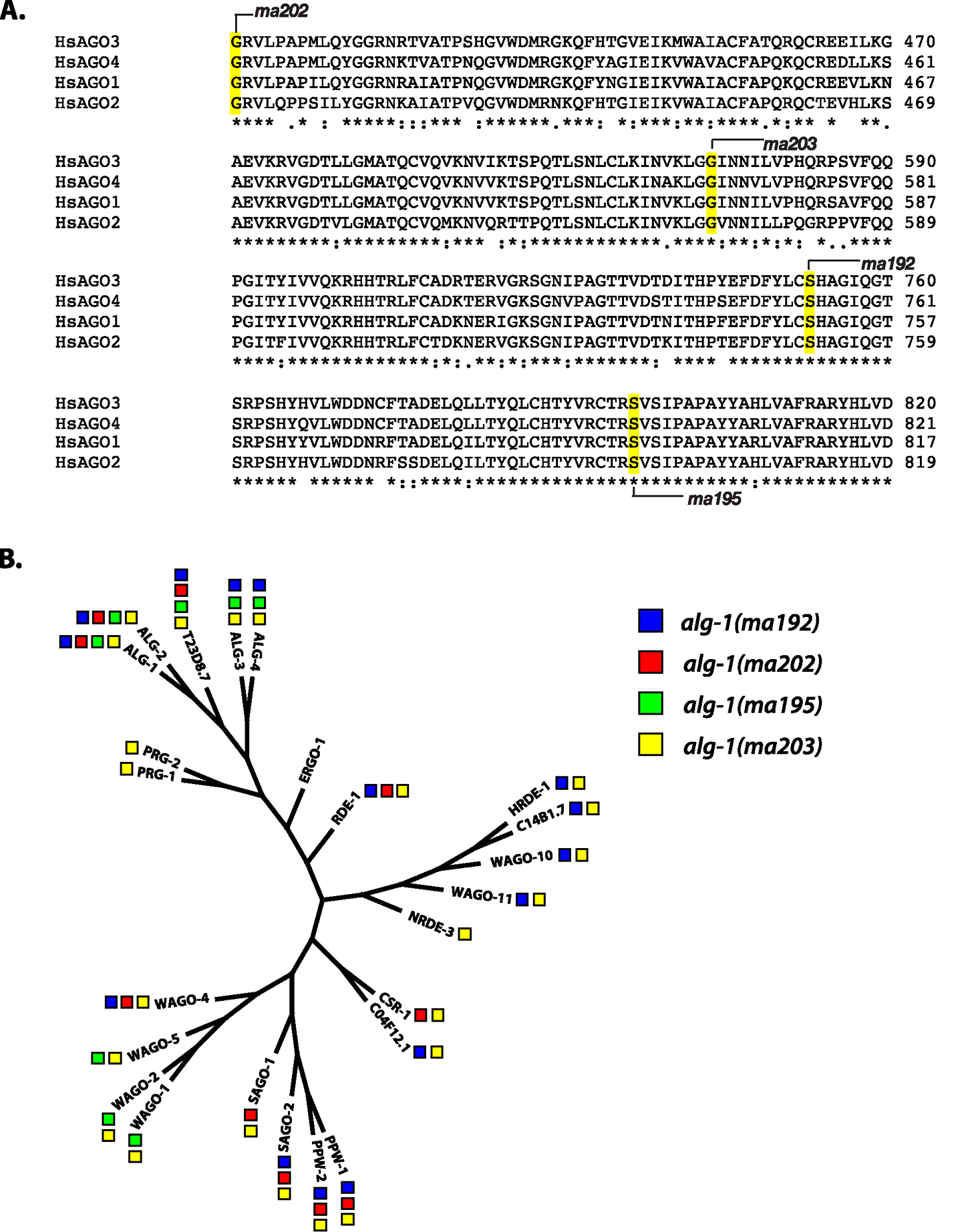

Supplement: Figure S1 — (A) Alignments of human AGO1-4 shows conservation of the amino acids affected by alg-1(anti) mutations. (B) A phylogenetic tree of C. elegans AGO proteins. Colored squares next to the AGO genes represent the presence of the amino acids affected by the alg-1(anti) mutations. Tree was created with Geneious using the nearest neighbor method. (TIF) [file pgen.1004286.s001.tif]

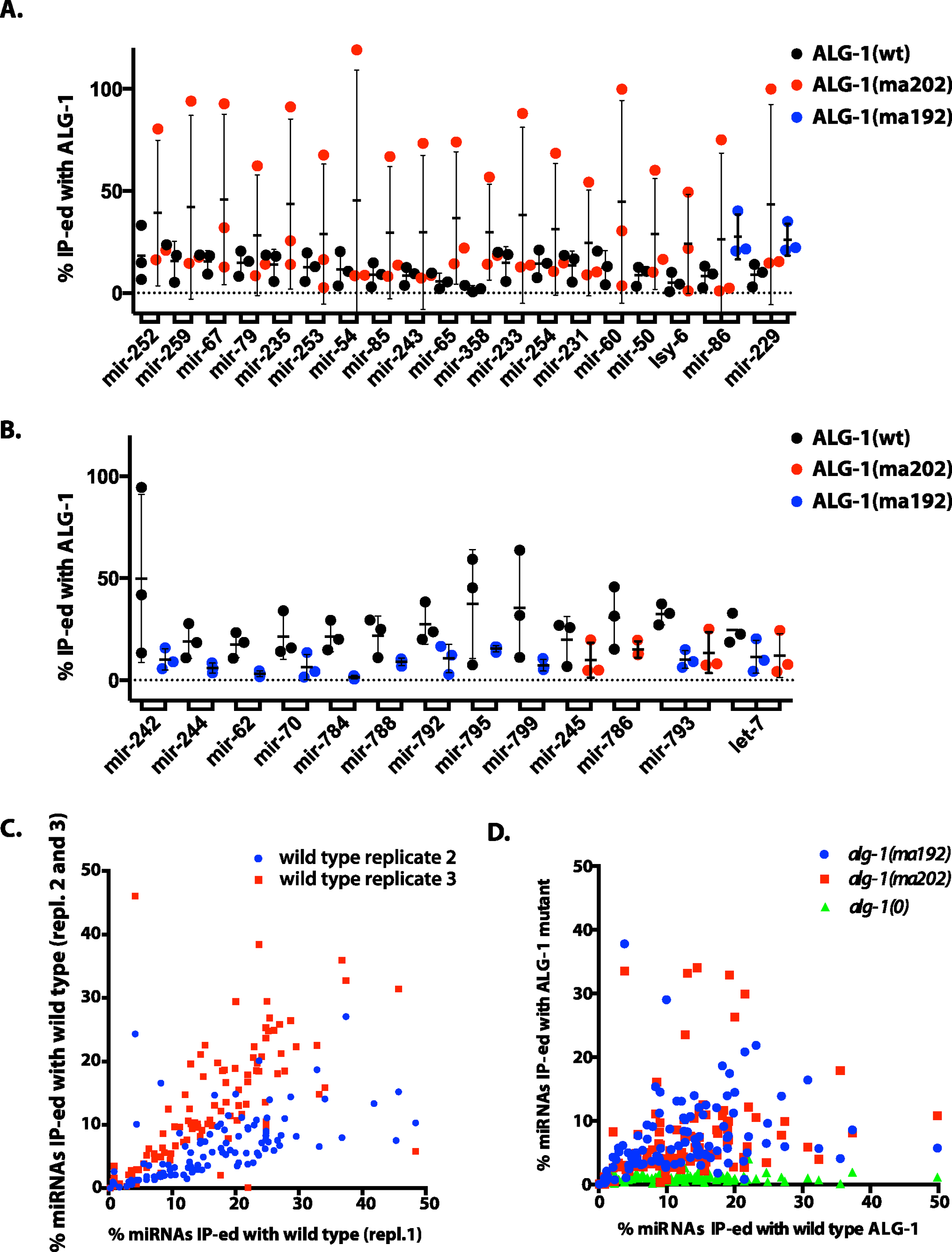

Supplement: Figure S2 — (A) A subset of microRNAs (highlighted by the oval in the top left quadrant of Figure 8E) that on average showed increased immunoprecipitation with ALG-1(anti) versus wild type ALG- and were immunoprecipitated at efficiencies of approximately 20% or more. Each dot represents a microRNA from a single biological replicate, all three replicates are plotted to show variability. (B) A subset of microRNAs (highlighted by the oval in the bottom right quadrant of Figure 8E) that on average showed a decreased association with ALG-1(anti) versus wild type ALG-1 and were immunoprecipitated at efficiencies of approximately 20% or more. Each dot represents a microRNA from a single biological replicate; all three replicates are plotted to show variability among replicates. (C) Scatterplot comparing microRNA association with wild type ALG-1 protein in three biological replicates. Technical variation between replicates results in imperfect correlation between the data sets. RNA isolated from ALG-1 immunoprecipitations was quantified using Taqman qRT-PCR. microRNA abundance in each IP was normalized to synthetic spike-in and to the amount of microRNA in the starting material. Data are plotted as % of microRNA levels in the starting material that had immunoprecipitated (IP-ed) with ALG-1. (D) Scatterplot comparing microRNA association with immunoprecipitated wild type (X-axis) and mutant (Y-axis) ALG-1. RNA isolated from ALG-1 immunoprecipitations was quantified using Taqman qRT-PCR. microRNA abundance in each IP was normalized to a synthetic spike-in and the amount of microRNA in the starting material, but not the amount of ALG-1 immunoprecipitated (IP-ed). Data are plotted as % of microRNA in the starting material that had IP-ed with wild type or mutant ALG-1. The graph shows average % IP-ed from 3 biological replicates. All strains carry lin-31(lf) and col-19::gfp in the background. lin-31 mutation is present in order to suppress alg-1(anti) vulval bursting phenotypes by non-heter [file pgen.1004286.s002.tif]

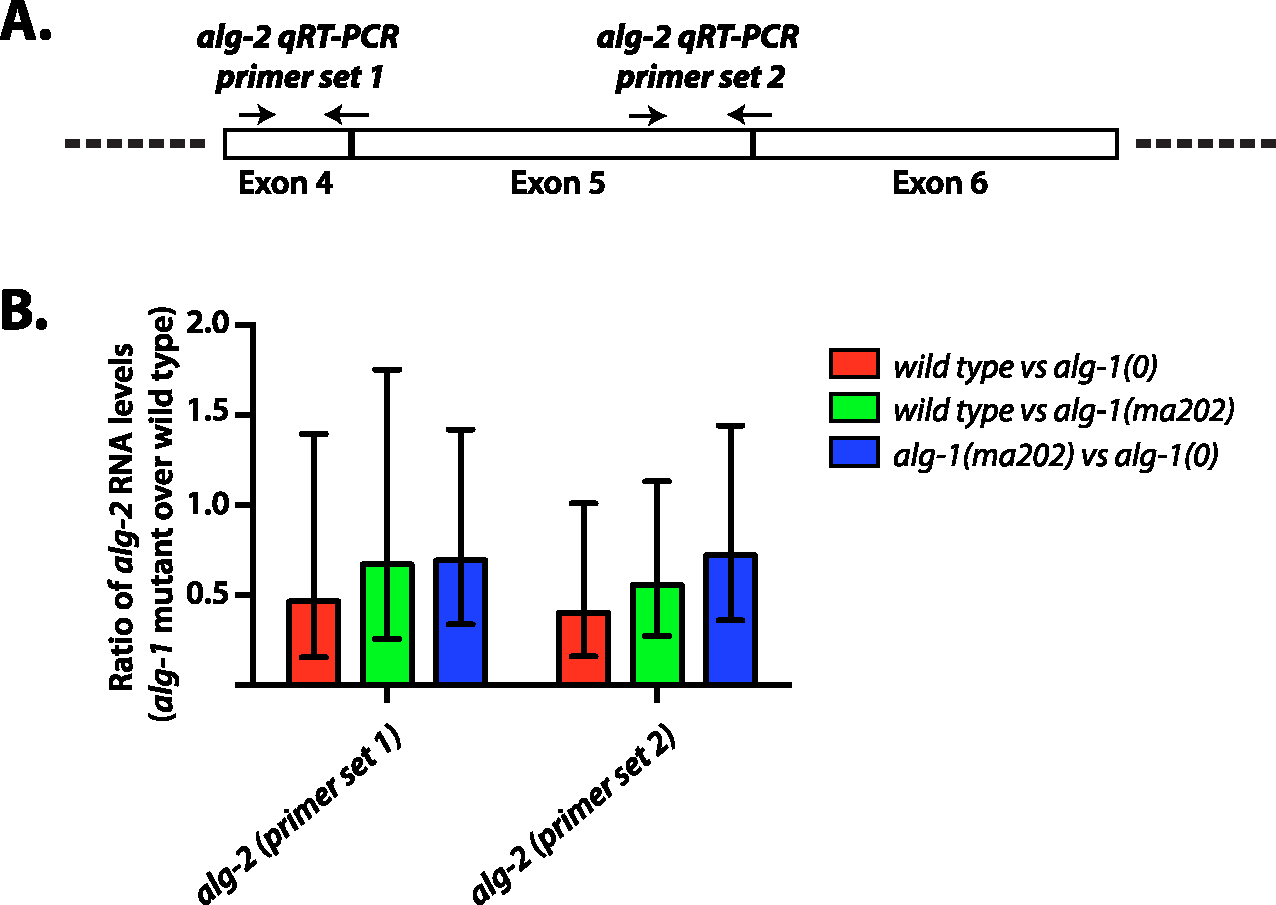

Supplement: Figure S3 — (A) A schematic showing positions of the two sets of RT-PCR primers within the alg-2 transcript. Only exons 4, 5, and 6 are drawn, with dash line representing the rest of the molecule. (B) Effects of alg-1 mutations on levels of alg-2 RNA as determined by qRT-PCR. Levels of alg-2 RNA are reduced approximately 2-fold in the alg-(0) mutants as compared to wild type, and less than 2-fold in alg-1(anti) mutants as compared to wild type. qRT-PCR was performed on RNA prepped from 3 biological replicates of alg-2(ok304) mixed stage animals using the Qiagen Quanti-fast kit. The results were normalized to tubulin RNA levels, and the ratio of alg-2 levels was calculated using the ΔΔCt method (Applied Biosystems). (TIF) [file pgen.1004286.s003.tif]
